# Genome-Wide Investigation of Pasteurella multocida Identifies the Stringent Response as a Negative Regulator of Hyaluronic Acid Capsule Production

**DOI:** 10.1128/spectrum.00195-22

**Published:** 2022-04-11

**Authors:** Thomas R. Smallman, Galain C. Williams, Marina Harper, John D. Boyce

**Affiliations:** a Infection and Immunity Program, Monash Biomedicine Discovery Institute and Department of Microbiology, Monash University, Melbourne, Victoria, Australia; Griffith University

**Keywords:** *Pasteurella multocida*, capsule, hyaluronic acid, stringent response, *P. multocida*

## Abstract

Pasteurella multocida is a Gram-negative capsulated bacterium responsible for a range of diseases that cause severe morbidity and mortality in livestock animals. The hyaluronic acid (HA) capsule produced by P. multocida serogroup A strains is a critical virulence factor. In this study, we utilized transposon-directed insertion site sequencing (TraDIS) to identify genes essential for *in vitro* growth of P. multocida and combined TraDIS with discontinuous density gradients (TraDISort) to identify genes required for HA capsule production and regulation in this pathogen. Analysis of mutants with a high cell density phenotype, indicative of the loss of extracellular capsule, led to the identification of 69 genes important for capsule production. These genes included all previously characterized genes in the capsule biosynthesis locus and *fis* and *hfq*, which encode known positive regulators of P. multocida capsule. Many of the other capsule-associated genes identified in this study were involved in regulation or activation of the stringent response, including *spoT* and *relA*, which encode proteins that regulate the concentration of guanosine alarmones. Disruption of the autoregulatory domains in the C-terminal half of SpoT using insertional mutagenesis resulted in reduced expression of capsule biosynthesis genes and an acapsular phenotype. Overall, these findings have greatly increased the understanding of hyaluronic acid capsule production and regulation in P. multocida.

**IMPORTANCE** The bacterial pathogen P. multocida can cause serious disease in production animals, including fowl cholera in poultry, hemorrhagic septicemia in cattle and buffalo, atrophic rhinitis in pigs, and respiratory diseases in a range of livestock. P. multocida produces a capsule that is essential for systemic disease, but the complete mechanisms underlying synthesis and regulation of capsule production are not fully elucidated. A whole-genome analysis using TraDIS was undertaken to identify genes essential for growth in rich media and to obtain a comprehensive characterization of capsule production. Many of the capsule-associated genes identified in this study were involved in the stringent response to stress, a novel finding for this important animal pathogen.

## INTRODUCTION

Pasteurella multocida is a Gram-negative coccobacillus that is the causative organism of serious animal diseases that collectively have a large economic impact worldwide. These diseases include fowl cholera in poultry and other birds, hemorrhagic septicemia in cattle and buffalo, atrophic rhinitis in pigs and rabbits, and upper and lower respiratory disease in cattle and other production animals (1). Self-limiting or chronic infections can be relatively mild, but many upper respiratory tract and systemic infections can be fatal within 1 to 2 days (1). P. multocida is highly transmissible, and large outbreaks of fowl cholera and hemorrhagic septicemia in both wild and domesticated animal populations have been reported (2, 3).

P. multocida is classified into five capsular serogroups, A, B, D, E, and F, with strains from each serogroup producing structurally distinct glycosaminoglycan capsule polymers (4, 5). The capsule biosynthesis locus representing each P. multocida capsule serogroup has been identified (6–8). Genes within each capsule locus encode proteins required for monomer synthesis and activation, glycosyltransferases required to synthesize the specific capsule polymer, and proteins involved in the ABC-transport system and in anchoring of the capsule to the outer membrane (4, 9–12). Directed mutation of capsule export genes in P. multocida strain VP161 (serogroup A) and strain M1404 (serogroup B) generated acapsular mutants that were unable to cause systemic disease in chickens and/or mice (13, 14), proving that capsule is an essential P. multocida virulence factor. In the serogroup A strain VP161, genes in the hyaluronic acid (HA) capsule biosynthesis locus are positively regulated by the nucleoid-associated regulatory protein Fis and the RNA chaperone Hfq (15, 16).

The importance of capsule for P. multocida virulence makes it an ideal target for novel therapeutics and vaccines. However, a complete understanding of the cellular signals that control capsule production in P. multocida and the regulatory mechanisms involved is lacking. In this study, we first utilized transposon-directed insertion site sequencing (TraDIS) to identify genes essential for P. multocida strain VP161 growth in rich media. We then phenotypically screened mutants using TraDISort to identify genes important for HA capsule production in strain VP161. The TraDISort method using density gradient centrifugation has been used previously to separate transposon mutants with altered cell density, which for capsulated bacteria is largely dependent on the amount of extracellular capsule present (17). Mutants with a higher or lower cell density relative to that of the majority of the mutant population are then analyzed by TraDIS to identify the inactivated gene in each mutant (17). In this study, we used TraDISort to isolate P. multocida VP161 transposon mutants with a higher cell density, indicative of reduced extracellular capsule, and identified a total of 69 genes important for capsule production in strain VP161. Individual mutants representing several genes identified by TraDISort analysis were confirmed to have reduced HA capsule production compared to that of the parent strain. Several of the genes associated with the acapsular phenotype were involved in the regulation or activation of the stringent response, which was previously not known to affect capsule production in this bacterium.

## RESULTS

### Generation of a saturated P. multocida strain VP161-Tn*7 Himar1* mutant library.

A kanamycin-resistant P. multocida strain, VP161-Tn*7*, was constructed (Table S1) to allow for use as a recipient in conjugation experiments with donor Escherichia coli strain AL2972 (Table S1), harboring the *Himar1* delivery vector pAL614 (18). Each conjugation mating generated more than 1 × 10^6^ P. multocida CFU when recovered on heart infusion (HI) media supplemented with kanamycin and spectinomycin. Approximately 6.45 × 10^6^ P. multocida *Himar1* transconjugants were pooled and stored in aliquots at −80°C for subsequent TraDIS experiments.

To identify genes that were essential for *in vitro* growth in HI and determine the quality and depth of the VP161-Tn*7 Himar1* library, two aliquots (A and B) of the mutant library were grown separately in HI medium (37°C, 200 rpm) to mid-exponential growth phase (optical density at 600 nm [OD_600_] of ∼0.5). DNA was isolated from cells harvested from each culture and used to generate TraDIS libraries; two different concentrations of adapter ligated material were used to ensure there was sufficient template for library amplification. Four TraDIS libraries were successfully amplified and sequenced on an Illumina MiSeq, namely, A1 and B1 (100 ng template) and A2 and B2 (250 ng template). The data were analyzed using the Bio-TraDIS pipeline to identify the total number of transposon-specific reads and the location of *Himar1* insertion sites in the VP161 genome (Fig. S1, Table S2). Comparison of unique *Himar1* insertion sites per gene gave high correlation coefficients between the rich media TraDIS library replicates regardless of the amount of DNA template used (Fig. S2), allowing the data to be combined and thus increasing the number of unique *Himar1* insertion sites across the genome and, therefore, the resolution. In total, 81,292 unique *Himar1* insertion sites were identified (Table S2), representing a unique insertion every 28 bp of the VP161 genome and an average of 38 unique insertions per gene. An insertion index was calculated for each gene by dividing the number of unique insertions by gene length, and insertion indexes for all genes were then used to generate two normally distributed data sets representing essential and nonessential genes (Fig. S3). Essential genes were determined to be those with an insertion index of less than 0.0134, defined as the intersection between the two curves. A total of 509 genes were identified as essential for P. multocida growth in HI broth (Table S3), encoding 473 predicted proteins, 35 tRNAs, and one 5S rRNA. Assignment of the encoded proteins to cluster of orthologous groups (COGs) revealed that a large proportion were predicted to be involved in translation, ribosomal structure and biogenesis. The 473 protein-encoding genes were compared to the database of essential genes (DEG) that contains genes identified as essential in other bacterial species (19). There were homologs in the DEG for 442 (93%) of the protein-encoding genes, indicating that TraDIS analysis of P. multocida grown *in vitro* in HI broth was a highly effective method to identify essential genes in this species (Table S3). Of the remaining 31 P. multocida essential genes encoding proteins, 17 encoded hypothetical proteins and 14 encoded proteins predicted to be involved in a range of processes, including the DNA repair and stress response, gene and protein regulation, nutrient synthesis, and contact-dependent growth inhibition (Table S3).

### Isolation of acapsular P. multocida cells via Percoll discontinuous density gradient centrifugation.

To separate mutants within the library based on cell density, TraDISort using consecutive Percoll discontinuous density gradients (10%, 40%, and 80% Percoll) was employed (Fig. S1 and Fig. S4A). Following the first centrifugation, the saturated P. multocida VP161-Tn*7 Himar1* mutant library separated into an upper, low-density cell layer containing capsulated cells and a lower, high-density cell layer (Fig. S4A). Mutants within the upper cell layer following this first centrifugation were not examined further, as they were predicted to produce wild-type levels of capsule. The lower layer of cells containing high-density cells was recovered, as this was predicted to contain cells with reduced extracellular capsule. A sample of this population was plated onto rich media. Following overnight growth, approximately 50% of the colonies generated were mucoid, indicating that a single gradient separation was insufficient to fully separate the acapsular cells from the cells capable of producing capsule. It is known that P. multocida cells in the exponential growth phase produce significantly more extracellular capsule than cells in other phases of growth (15). To allow transient acapsular mutants the opportunity to produce extracellular capsule, the lower cell layer population was grown for 4 h in HI broth. The cells were recovered and then subjected to a second Percoll discontinuous density gradient. Following centrifugation, the cells separated into a top, low-density (termed LD2) cell layer and a bottom, high-density (termed HD2) cell layer. Each population was recovered and plated separately onto HI agar plates. All colonies generated from cells recovered from the LD2 cell layer displayed a capsulated phenotype (large, mucoid), and approximately 99% of the colonies generated from cells recovered from the HD2 layer displayed an acapsular phenotype (small, nonmucoid), confirming that consecutive density gradient centrifugations were required to successfully separate high-density mutants with an acapsular phenotype.

To confirm that mutants producing small and nonmucoid colonies represented acapsular mutants, genomic DNA was isolated from 35 nonmucoid colonies recovered from the HD2 cell layer and used separately as the template in a Sanger sequencing reaction with an outward-firing, *Himar1*-specific primer (BAP3453, Table S4) to identify the transposon insertion site in the genome of each mutant. The majority of the 35 *Himar1* mutants examined had insertions within genes known to be required for capsule biosynthesis. Interestingly, several genes not previously known to be involved in capsule production were also identified, including *pgm*, *ppx*, and *cpxA*. Absorbance assays measuring the amount of extracellular HA capsular material produced by *Himar1* mutants representing eight genes showed that all produced significantly reduced amounts of HA compared to the parent strain, VP161-Tn*7* (Fig. S4B). Together, these data show that consecutive discontinuous Percoll density gradients were successful in isolating acapsular mutants from the large VP161-Tn*7 Himar1* mutant library.

### Identification of capsule-associated genes in VP161.

**FIG 1:**
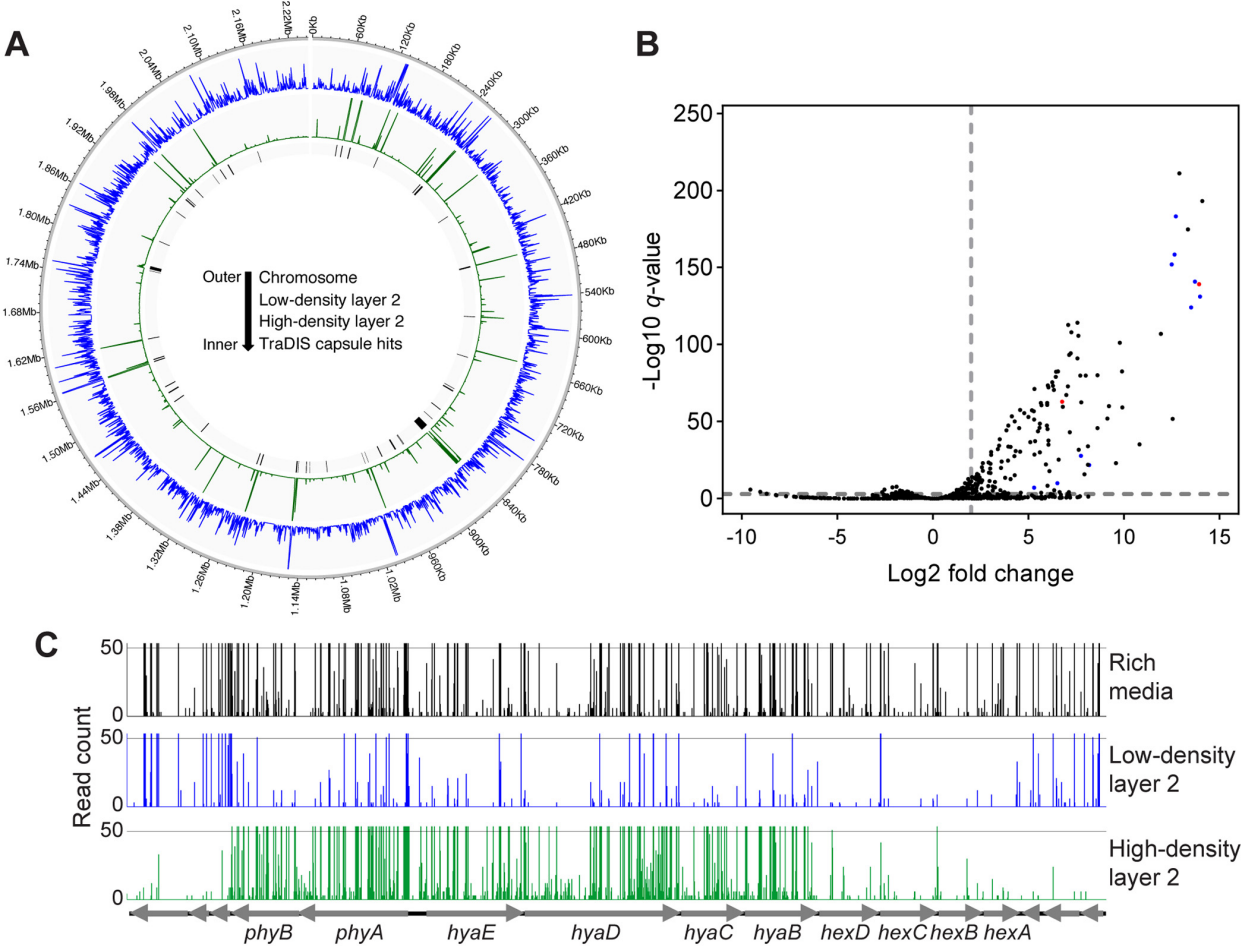
Overview of TraDISort library data. (A) The number of *Himar1* insertions per 500 bp across the VP161 genome in the TraDIS libraries generated using either the top, LD2 cell layer (predominantly capsulated cells, insertions sites in blue) or the bottom, HD2 cell layer (predominantly acapsular, insertion sites in green). Cell layers LD2 and HD2 were recovered from the second discontinuous Percoll gradient centrifugation. Genes identified as important for P. multocida hyaluronic acid (HA) capsule production are indicated using black bars. (B) Volcano plot showing the log_2_ fold change in the number of *Himar1* insertions per gene in the TraDIS libraries generated using the HD2 cell layer compared to TraDIS libraries generated using the LD2 cell layer following consecutive discontinuous Percoll gradient centrifugations, with the −log_10_ *q* value provided for each comparison. Genes in the HD2 cell layer with a log_2_ fold change of >2 and a *q* value of <0.001 increase in *Himar1* insertions compared to those in the LD2 cell layer were identified as important for capsule production (both cutoffs shown as gray dotted lines). Known capsule biosynthesis genes are shown in blue, and known positive regulators of HA capsule production are shown in red. (C) TraDIS library analysis showing number of *Himar1* insertions mapping to the VP161 capsule locus in the input *Himar1* library (black) and after consecutive discontinuous Percoll gradients; top, LD2 cell layer (capsulated, blue), and bottom, HD2 cell layer (acapsular, green). The majority of mutants with *Himar1* insertions in the capsule biosynthesis locus separated into the HD2 cell layer of the Percoll gradient, as shown by data showing a large increase in *Himar1* insertions in these genes in mutants recovered from the bottom, HD2 cell layer compared to the insertions in the same genes from mutants recovered from the top, LD2 cell layer.

Genomic DNA was extracted from the population of VP161-Tn*7 Himar1* mutants recovered from the top, LD2 cell layer (predicted to be capsulated) and separately from the population of mutants in the bottom, HD2 cell layer (predicted to be acapsular). Separate TraDIS libraries were generated using genomic DNA from both cell layers, sequenced on an Illumina MiSeq, and analyzed by Bio-TraDIS and modified scripts (Fig. S1). The number of unique *Himar1* insertions was highly correlated between the LD2 and HD2 library replicates (Fig. S5A), and principal-component analysis of unique *Himar1* insertions and total insertions per gene showed that HD2 library replicates clustered away from LD2 and rich media libraries (Fig. S5B). Whole-genome *Himar1* insertion density plots generated from the LD2 layer TraDIS libraries showed that the *Himar1* insertion counts were distributed relatively evenly across the VP161 genome (Fig. 1A). In contrast, *Himar1* insertions generated from sequences derived from the HD2 cell layer (predominantly an acapsular population) showed that the *Himar1* insertions were concentrated in only a few genomic regions (Fig. 1A). As acapsular mutants were enriched in the HD2 layer, any gene that had a >4.0-fold increase (>2.0 log2-fold increase) in *Himar1* insertions in the HD2 layer compared to those in the LD2 layer (*q* value of <0.001, insertion index of >0.8) was identified as important for HA capsule production in strain VP161 (Fig. 1B). This analysis identified 69 genes important for HA capsule production, including all genes in the capsule biosynthesis locus (Table 1, Fig. 1C), *fis* and *hfq*, which encode known positive regulators of capsule biosynthesis genes, and the novel capsule-associated genes identified by Sanger sequencing of individual mutants, namely, *pgm*, *ppx*, and *cpxA*. These data confirm that the use of two consecutive discontinuous density gradient centrifugations had successfully isolated acapsular mutants from the saturated VP161-Tn*7 Himar1* library. Other genes that had a significant increase in the number of transposon insertions in this population of mutants were involved in a wide range of functions, including the stringent response, central metabolism and carbohydrate biosynthesis, and translation, transport, and ATP synthesis (Table 1). Interestingly, except for *fis*, no other genes encoding transcription factors were identified, suggesting that there are no other transcription factors that act as direct positive regulators of HA capsule biosynthesis in strain VP161.

**TABLE 1:** Genes identified using TraDISort as important for hyaluronic acid capsule production in P. multocida strain VP161

Gene name	VP161 locus tag	Predicted function	Log_2_ fold change	*q* value	Insertion index ratio
PmVP161_0059	PmVP161_0059	Hypothetical protein	7.6	2.78E−106	1.1
*yibF*	PmVP161_0060	Putative GST-like protein YibF	2.3	1.46E−07	0.9
*dus*	PmVP161_0075	Putative tRNA-dihydrouridine synthase	14.1	6.77E−194	11.4
*fis*	PmVP161_0076	DNA-binding protein Fis	13.9	5.74E−140	14.0
*pgm*	PmVP161_0089	Phosphoglucomutase	12.9	1.05E−211	4.8
*cpdA*	PmVP161_0148	3′,5′-Cyclic adenosine monophosphate phosphodiesterase CpdA	8.1	1.08E−22	2.1
*znuA*	PmVP161_0255	High-affinity zinc uptake system protein ZnuA	7.2	1.02E−43	3.1
*gmk*	PmVP161_0260	Guanylate kinase	7.1	0.000618	3.0
*rpoZ*	PmVP161_0261	DNA-directed RNA polymerase subunit omega	6.8	3.22E−60	1.5
*spoT*	PmVP161_0262	Bifunctional (p)ppGpp synthase/hydrolase SpoT	6.0	1.19E−35	1.7
*seqA*	PmVP161_0336	Negative modulator of initiation of replication	5.6	2.22E−49	0.9
*glnD*	PmVP161_0442	Bifunctional uridylyltransferase/uridylyl-removing enzyme	7.6	8.78E−115	0.9
*prmC*	PmVP161_0549	Release factor glutamine methyltransferase	12.5	2.13E−52	26.0
*truA*	PmVP161_0625	tRNA pseudouridine synthase A	4.1	3.03E−34	0.8
*ubiX*	PmVP161_0693	Flavin prenyltransferase UbiX	3.9	3.79E−25	0.9
*pta*	PmVP161_0698	Phosphate acetyltransferase	6.3	4.42E−06	1.6
*dnaJ*	PmVP161_0735	Chaperone protein DnaJ	9.1	1.50E−52	3.5
*rimP*	PmVP161_0757	Ribosome maturation factor RimP	9.9	2.84E−83	2.7
*phyB*	PmVP161_0773	Capsule phospholipid substitution protein	12.7	5.07E−159	6.1
*phyA*	PmVP161_0774	Capsule phospholipid substitution protein	12.5	1.16E−152	6.2
*hyaE*	PmVP161_0775	Hyaluronic acid capsule biosynthesis protein	13.7	1.42E−141	6.0
*hyaD*	PmVP161_0776	Hyaluronic acid synthase	14.0	1.03E−131	8.4
*hyaC*	PmVP161_0777	UDP-glucose 6-dehydrogenase	13.5	8.45E−125	12.2
*hyaB*	PmVP161_0778	Hyaluronic acid capsule biosynthesis protein	12.7	5.64E−184	5.8
*hexD*	PmVP161_0779	Hyaluronic acid capsule export	8.1	1.97E−22	3.7
*hexC*	PmVP161_0780	Hyaluronic acid capsule export	7.8	2.08E−28	3.2
*hexB*	PmVP161_0781	Hyaluronic acid capsule transport protein HexB	6.5	1.34E−10	2.2
*hexA*	PmVP161_0782	Hyaluronic acid capsule transport ATP-binding protein HexA	5.3	7.72E−08	2.0
PmVP161_0825	PmVP161_0825	Hypothetical protein	6.3	4.00E−76	0.9
*holC*	PmVP161_0828	DNA polymerase III subunit chi	2.9	7.33E−09	1.5
*gyrA*	PmVP161_0847	DNA gyrase subunit A	9.6	1.19E−23	3.0
PmVP161_0878	PmVP161_0878	Hypothetical protein	11.9	1.36E−107	18.4
*ptsH*	PmVP161_0905	Phosphocarrier protein HPr	9.8	6.26E−102	2.9
*hfq*	PmVP161_0914	RNA-binding protein Hfq	6.8	1.61E−63	2.5
*znuB*	PmVP161_0986	High-affinity zinc uptake system membrane protein ZnuB	6.0	5.09E−09	1.1
*secG*	PmVP161_1023	Protein export membrane protein SecG	10.8	7.65E−36	7.0
*epmA*	PmVP161_1029	Elongation factor P-(R)-beta-lysine ligase	5.7	2.78E−61	0.9
*queD*	PmVP161_1046	6-Carboxy-5,6,7,8-tetrahydropterin synthase	7.1	1.63E−73	1.0
*rnb*	PmVP161_1050	Exoribonuclease 2	5.5	2.24E−44	1.2
*efp*	PmVP161_1135	Elongation factor P	7.9	1.95E−16	7.0
*epmB*	PmVP161_1136	l-Lysine 2,3-aminomutase	7.6	2.12E−32	1.7
*alsT_2*	PmVP161_1144	Amino acid carrier protein last	4.6	4.65E−31	1.1
*rseP*	PmVP161_1260	Regulator of sigma-E protease RseP	5.7	0.00018	1.6
*cpxA*	PmVP161_1346	Sensor histidine kinase CpxA	2.5	1.94E−14	0.9
*relA*	PmVP161_1370	GTP pyrophosphokinase	6.3	6.54E−74	1.1
*purB*	PmVP161_1383	Adenylosuccinate lyase	4.0	4.34E−48	0.9
*ppx*	PmVP161_1426	Exopolyphosphatase	8.6	9.75E−81	2.2
*trmA*	PmVP161_1430	tRNA/tmRNA [uracil-C(5)]-methyltransferase	7.6	8.83E−92	1.2
PmVP161_1431	PmVP161_1431	Hypothetical protein	9.2	1.14E−60	2.5
*tufA_1*	PmVP161_1480	Elongation factor Tu 1	6.0	1.33E−61	1.5
*mioC*	PmVP161_1625	Protein MioC	3.0	0.000615	0.9
PmVP161_1628	PmVP161_1628	Hypothetical protein	7.1	7.11E−94	2.2
*atpB*	PmVP161_1629	ATP synthase subunit a	4.8	3.22E−56	4.5
*atpH_1*	PmVP161_1630	ATP synthase subunit c	7.8	3.43E−63	2.1
*atpF*	PmVP161_1631	ATP synthase subunit b	8.1	1.32E−34	3.5
*atpH_2*	PmVP161_1632	ATP synthase subunit delta	4.3	1.10E−50	2.2
*atpA*	PmVP161_1633	ATP synthase subunit alpha	8.0	1.42E−80	2.5
*atpG*	PmVP161_1634	ATP synthase gamma chain	7.0	5.52E−68	2.9
*atpD*	PmVP161_1635	ATP synthase subunit beta	7.7	1.86E−80	2.2
*pgl*	PmVP161_1693	6-Phosphogluconolactonase	5.6	1.24E−30	1.0
*rpe*	PmVP161_1767	Ribulose-phosphate 3-epimerase	3.8	4.88E−45	1.2
*thiE*	PmVP161_1798	Thiamine-phosphate synthase	4.0	0.000551	0.9
*thiM*	PmVP161_1800	Hydroxyethylthiazole kinase	6.6	6.31E−05	2.2
*ssuB*	PmVP161_1804	Aliphatic sulfonates import ATP-binding protein SsuB	6.6	5.96E−26	2.5
*galU*	PmVP161_1828	UTP-glucose−1-phosphate uridylyltransferase	13.3	2.19E−175	5.4
PmVP161_1879	PmVP161_1879	Hypothetical protein	7.1	7.15E−05	1.5
*tufA_2*	PmVP161_1902	Elongation factor Tu 1	6.0	9.15E−63	1.4
*glmS*	PmVP161_1917	Glutamine-fructose-6-phosphate aminotransferase (isomerizing)	5.9	6.37E−23	1.6
*rpiA*	PmVP161_1987	Ribose-5-phosphate isomerase A	8.6	2.36E−46	2.7

### Validation of novel capsule production genes.

**FIG 2:**
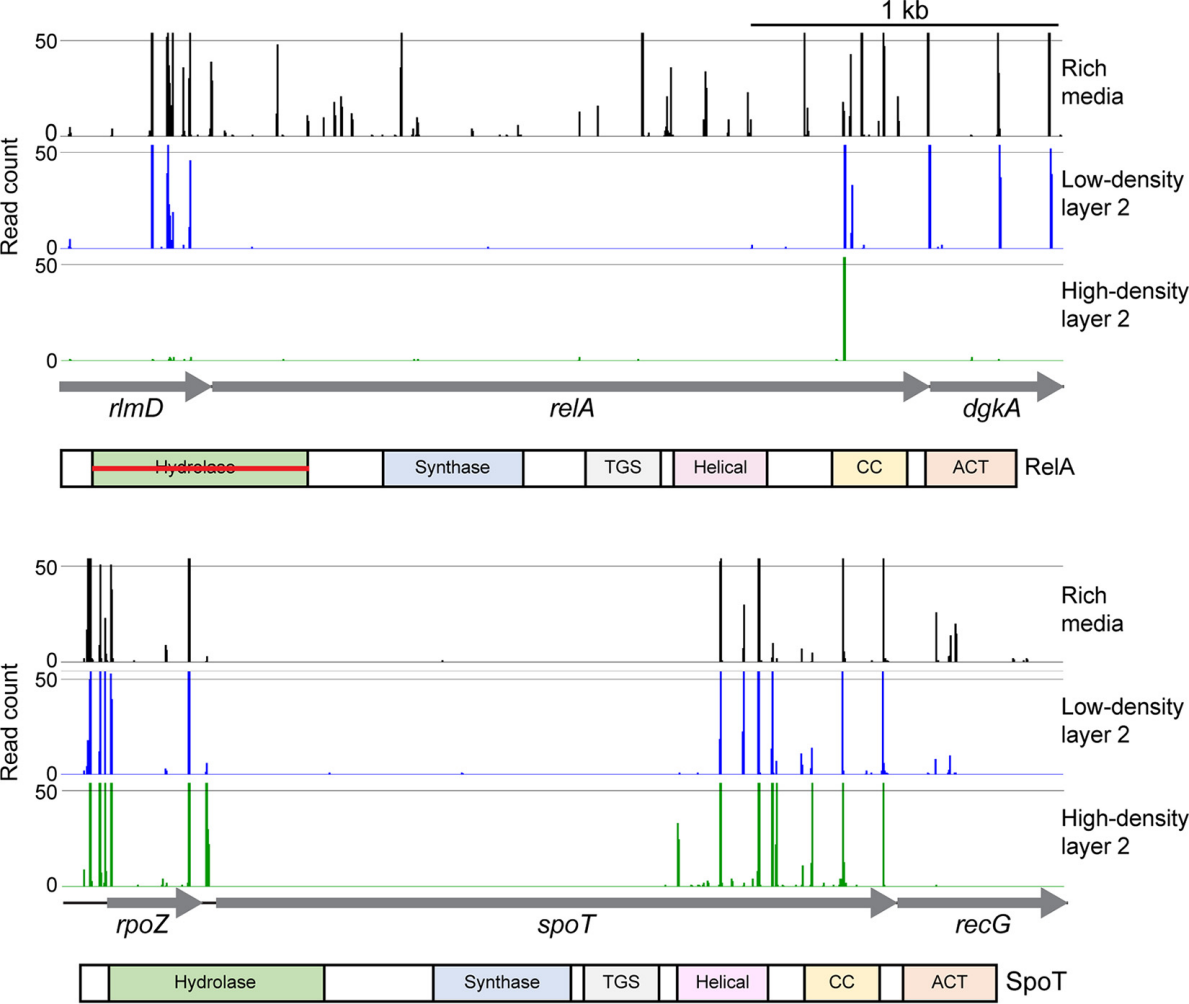
Insertion site plots showing *Himar1* insertions in *spoT* and *relA*. Insertion sites represent combined data from the rich media TraDIS libraries (black), LD2 cell layer TraDIS libraries (blue), and HD2 cell layer TraDIS libraries (green). Below the insertion site plots is a graphical representation of domains in RelA and SpoT. TGS, ThrRS GTPase domain; CC, coiled coil/zinc finger domain; ACT, aspartokinase, chorismate mutase domain. The horizontal red line in the RelA Hydrolase domain indicates that this domain is predicted to be non-functional based on similarity to the E. coli homolog.

To confirm that the genes newly identified using TraDISort were important for HA capsule production, TargeTron-directed mutagenesis of a number of genes was attempted. In total, three TargeTron mutants were successfully constructed; these mutants had insertions in *ppx* and *ptsH* and in the 3′ regulatory region of *spoT* (Table S1). The 3′ region of *spoT* was chosen for TargeTron insertion, as TraDISort analysis indicated that the majority of HD2 *spoT* mutants had insertions in this region (Fig. 2). To add to these validation experiments, *pgm* and *galU Himar1* mutants isolated from the HD2 cell layer were also included. Each mutant was confirmed to have a single transposon insertion by Sanger sequencing as described above.

**FIG 3:**
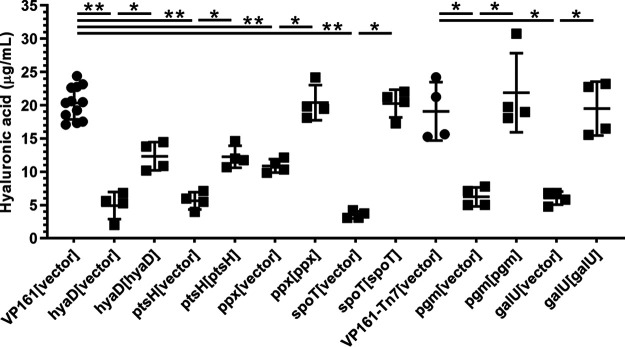
Amount of hyaluronic acid (HA) capsule produced by P. multocida parent strains VP161 and VP161-Tn*7*, VP161 TargeTron mutants (*hyaD*, *ptsH*, *ppx*, *spoT*), or *Himar1* mutants (*pgm*, *galU*). Each mutant was provided with the appropriate vector (pAL99S or pAL99T) or an intact copy of the target gene (provided on the appropriate vector) to demonstrate that the gene inactivated in each strain was responsible for the acapsular phenotype. HA production by each strain was measured in biological quadruplicate using mid-exponential-phase growth cultures. Significant differences in the amount of capsule produced by different strains were determined using Mann-Whitney U test; *, *P < *0.05; **, *P < *0.01. Error bars represent mean ± standard deviation (SD).

**FIG 4:**
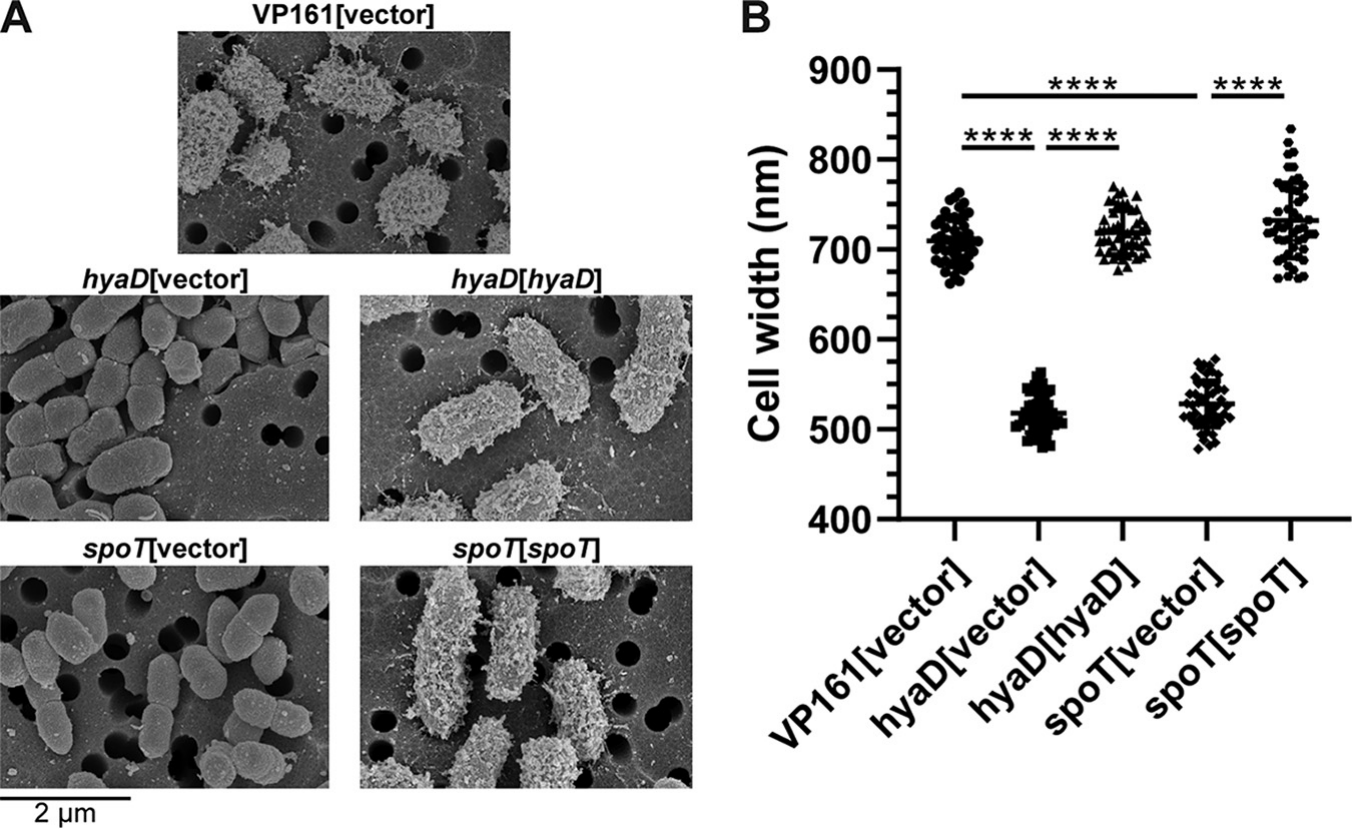
Scanning electron microscopy (SEM) of P. multocida. The parent strain VP161 was provided with pAL99S vector, and the VP161 3′ *spoT* and *hyaD* TargeTron mutants were provided with the pAL99S vector or with an intact copy of the appropriate gene on pAL99S (complemented mutant). SEM was performed using mid-exponential-phase growth cultures. (A) Electron micrographs of each strain showing the smooth surface of the 3′ *spoT* and *hyaD* mutant strains containing vector only (acapsular cells) and the ruffled surface of the parent and complemented mutant strains (capsulated cells). (B) Cell widths of 50 individual cells of each strain as measured using the FIJI imaging package in ImageJ. Significant differences in width (lower values indicative of reduced surface capsule) were determined using an unpaired *t* test. ****, *P < *0.0001; error bars, mean ± SD.

To prove that the inactivated gene in each mutant was responsible for the acapsular phenotype, complementation experiments were conducted. The appropriate complementation plasmid and corresponding empty vector were used separately to transform each mutant to generate complemented mutant and empty vector control strains (Table S1). The amount of HA capsule produced by each of the mutant strains harboring empty vector and the corresponding complemented mutant was determined by HA absorbance assays using cells grown to mid-exponential growth phase. The amount of HA produced by each strain was compared to HA production in the corresponding parent strain harboring empty vector (VP161 for TargeTron mutants and VP171-Tn*7* for *Himar1* mutants). A VP161 *hyaD* TargeTron mutant (Table S1) was included as a known acapsular control strain. As expected, all mutants harboring empty vector produced significantly less HA than the parent strains VP161 and VP161-Tn*7* (Fig. 3). All mutants provided with an intact copy of the appropriate gene in *trans* produced significantly more HA than the corresponding mutant harboring empty vector, although the amount of HA was not restored to the same level as the parent strain for all mutants (Fig. 3). Scanning electron microscopy (SEM) of the *spoT* TargeTron mutant was performed to show that loss of HA capsular material correlated with less capsule on the surface of the bacterium. For this experiment, the parent strain, VP161, and the VP161 *hyaD* TargeTron mutant were used as known capsular and acapsular controls, respectively. Imaging of cells in the mid-exponential phase of growth revealed that the wild-type VP161 cells had a ruffled outer surface indicative of a capsule layer that was absent in both the *hyaD* and *spoT* mutants harboring empty vector but was present in the complemented mutant strains (Fig. 4). Corresponding to less capsule, both the *hyaD* and *spoT* mutants had significantly reduced cell widths compared to wild-type VP161, and mutant cells provided with the appropriate complementation plasmid had a width similar to that of wild-type VP161 cells (Fig. 4). Together, these data show that novel capsule-associated genes identified by TraDIS were required for HA capsule production in VP161.

### Disruption of the 3′ regulatory region of *spoT* results in reduced expression of the capsule biosynthesis genes independent of *fis*.

The proteins encoded by *spoT* and *relA* are homologs of proteins within the RelA/SpoT homolog (RSH) superfamily. In our study, TraDISort *spoT* and *relA* mutants enriched in the HD2 cell population had insertions predominantly in the 3′ region of the gene (Fig. 2). In E. coli, RSH proteins play central roles in the stringent response to various stresses by controlling the levels of the guanosine alarmone molecules 5′-diphosphate-3′-diphosphate and GTP-3′-diphosphate [collectively named (p)ppGpp]. RelA is a (p)ppGpp synthase that in other bacteria has been shown to respond to amino acid starvation. SpoT is a bifunctional (p)ppGpp synthase/hydrolase that in E. coli has been shown to respond to iron, phosphate, and fatty acid starvation (20–23). The C-terminal regulatory region of both proteins contains four domains, namely, the ThrRS, GTPase, and SpoT (TGS) domain, the helical domain, the conserved cysteine or zinc finger domain (CC/ZFD), and the aspartokinase, chorismate mutase, and TyrA (ACT) domain (Fig. 2) (24). There were transposon insertions across the entire *relA* gene following *in vitro* growth in HI, including the N-terminal domain encoding the synthase domain, indicating that RelA (p)ppGpp synthase activity is not essential for normal growth *in vitro* in rich media (Fig. 2). However, the TraDISort read data revealed that there was only a very small number of transposon sites in the *relA* mutant population recovered from the second gradient centrifugation, and these were predominantly in the 3′ regulatory region. This indicates that the majority of *relA* mutants present in the input library had a cell density different from that of both the LD2 and HD2 population and were therefore lost from the TraDISort analysis. However, the majority of the *relA* mutants still present in the HD2 cell layer (log_2_ fold change increase of 6.3 compared to those in the LD2 cell layer) had *Himar1* insertion sites predominantly at nucleotide 1975 or nucleotide 1976, both located upstream of the region encoding the ACT domain.

The 5′ region of *spoT*, encoding the (p)ppGpp hydrolase and synthase domains, was deemed essential for the growth of P. multocida *in vitro* in HI media, as no mutants with transposon insertions in this region were recovered following growth *in vitro* in HI media (Fig. 2). However, there were a significant number of mutants with transposon insertions throughout the 3′ regulatory region of *spoT* (Fig. 2). TraDISort analysis revealed that although some of the 3′ *spoT* mutants were present in the LD2 cell layer, most were present in the HD2 cell layer (log_2_ fold change increase of 6.0 in the HD2 cell layer compared to the LD2 cell layer), correlating with the acapsular phenotype displayed by the 3′ *spoT* TargeTron mutant. The *Himar1* transposon insertions in the HD2 mutants were distributed throughout the 3′ regulatory region between nucleotides 1416 to 2124 but particularly in the region encoding the helical domain, the CC/ZFD domain, and the ACT domain (Fig. 2).

**FIG 5:**
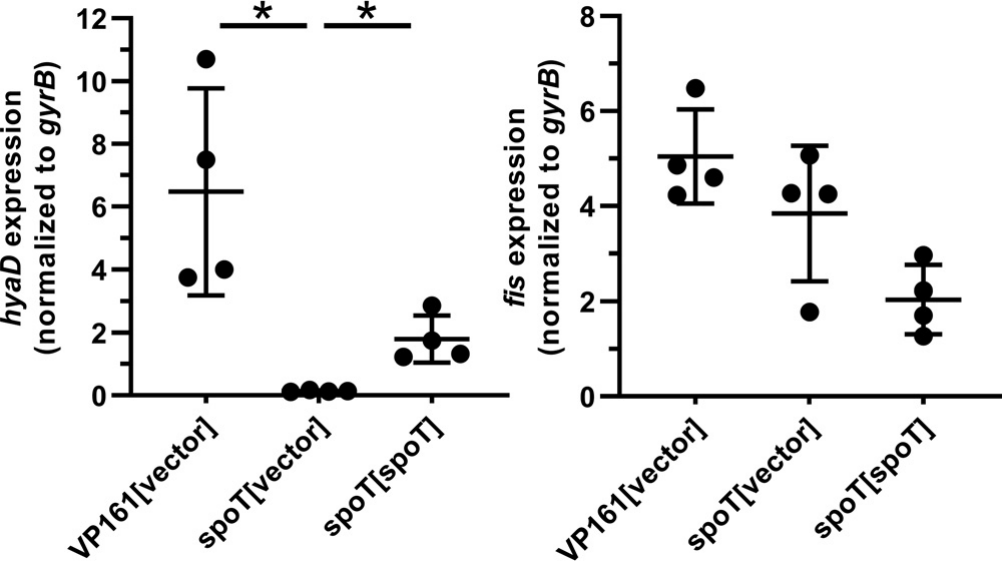
Relative expression of the capsule-specific glycosyltransferase gene, *hyaD*, and the global regulator gene, *fis*, in the P. multocida 3′ *spoT* mutant. Fis is known to positively regulate the expression of the capsule genes in P. multocida VP161 (16). Using qRT-PCR, we measured the expression of each gene in the VP161 parent strain containing empty vector and VP161 *spoT* mutant (with empty vector) and the complemented mutant. Expression levels were measured in biological quadruplicate using RNA extracted from mid-exponential growth phase cultures. Expression of both genes was normalized to the level of housekeeping gene *gyrB*. Error bars represent the mean ± standard deviation. Gene expression levels between strains were compared using a Mann-Whitney U test. *, *P* < 0.05, mean ± SD.

Disrupting the 3′ regulatory region of *relA* in E. coli, or the equivalent region in the *spoT* homolog *rel* from Mycobacterium smegmatis and Streptococcus equisimilis, results in increased (p)ppGpp synthesis and/or decreased (p)ppGpp hydrolysis by the modified proteins (25–28). Moreover, increased concentration of ppGpp in E. coli results in downregulation of the gene encoding the nucleoid-associated regulatory protein Fis (29, 30). In P. multocida, Fis positively regulates the expression of genes within the capsule biosynthesis, and in the absence of Fis, the bacterium is unable to produce HA capsule (16). To determine if the P. multocida 3′ *spoT* TargeTron mutant (AL3391, Table S1) with an acapsular phenotype also had reduced expression of *fis*, quantitative reverse transcriptase PCR (qRT-PCR) was performed to measure the expression of *fis* and capsule biosynthesis gene *hyaD* relative to the expression of the housekeeping gene *gyrB*. Expression of these genes during mid-exponential-phase growth was measured in wild-type VP161 harboring empty vector, the *spoT* TargeTron mutant harboring empty vector, and the complemented *spoT* mutant. The *spoT* mutant harboring empty vector had significantly reduced *hyaD* expression compared to VP161, and expression of this gene was partially restored when the mutant was provided with an intact copy of *hyaD* in *trans* (Fig. 5). However, there was no significant difference in *fis* expression between these strains (Fig. 5), indicating that disrupting the C-terminal regulatory region of SpoT resulted in decreased capsule biosynthesis gene expression, but this regulatory change was independent of Fis.

## DISCUSSION

In this study, we first used TraDIS analysis of a saturated transposon library to identify genes that were essential for *in vitro* growth of P. multocida strain VP161 in rich media. Following the identification of essential genes, we used TraDISort to identify genes that when inactivated were associated with a high-density, acapsular cell phenotype. Capsule is an essential virulence factor, as it allows P. multocida to avoid complement-mediated killing and phagocytosis (13, 14).

TraDIS analysis identified 509 genes as essential for growth of strain VP161 in rich media, which was similar to the number of essential genes identified using transposon insertion sequencing methods in other bacterial species (19). Moreover, approximately 93% of the 473 P. multocida proteins identified as essential for growth in rich media were homologs of essential proteins in another bacterial species, the majority of which were also identified using cells grown *in vitro* in rich media. Thus, our TraDIS analysis successfully identified genes truly essential for P. multocida growth in rich media. Most of the essential P. multocida genes encoded proteins involved in housekeeping processes, with a large proportion involved in translation and cell wall biosynthesis. Essential genes included those required for glycolysis, pyruvate oxidation, and gluconeogenesis, for the biosynthesis of membranes and cell wall components (phosphatidylethanolamine, lipid A, peptidoglycan), for the synthesis of nucleotides, lysine, vitamins, and cofactors, and for housekeeping processes such as ribosome production, tRNA synthetases, DNA replication, cell division, and Sec protein export. Genes in several of these pathways have also been identified in other bacterial species as essential for growth in rich media (31–34).

Following essential gene analysis, TraDISort was used to identify genes important for HA capsule production. Consecutive Percoll discontinuous gradient centrifugations were employed to isolate mutants with a high-density, acapsular phenotype. Genes identified as inactivated in these mutants included all genes within the capsule biosynthesis locus as well as genes encoding the global regulator Fis and the Hfq RNA chaperone that each play a role in the positive regulation of capsule in P. multocida serogroup A strains (15, 16). Five genes identified as important for capsule production were investigated further. Disrupting *ppx*, *ptsH*, *galU*, *pgm*, and the 3′ autoregulatory region of *spoT* resulted in reduced HA capsule production that was restored when each mutant was provided with the appropriate gene in *trans*. Moreover, SEM of the VP161 3′ *spoT* mutant and the complemented mutant demonstrated a clear correlation between inactivation of the regulatory region of SpoT and an acapsular phenotype.

Several genes that were identified as important for HA capsule production encoded proteins involved in carbohydrate metabolism and the production of HA capsule monomers UDP-*N*-acetyl-d-glucosamine (UDP-GlcNAc) and UDP-glucuronic acid (UDP-GlcA). The genes *pgm* and *galU* encode homologs of phosphoglucomutase Pgm and glucose-1-phosphate uridylyltransferase GalU, which together with the UDP-glucose dehydrogenase HyaC synthesize UDP-GlcA from glucose (9, 35, 36). Both *pgm* and *galU* are required for UDP-GlcA synthesis and capsule production in Streptococcus pneumoniae (serogroups 1 and 3) and in Klebsiella pneumoniae strain NTUH-K2044 (17, 37, 38). In P. multocida, we predict that inactivation of *pgm* or *galU* would stop UDP-GlcA synthesis and hence stall HA capsule production. Enriched in the HD2 cell layer were mutants with insertions in *glmS*, encoding l-glutamine–d-fructose-6-phosphate aminotransferase, which converts β-d-fructose 6-phosphate to α-d-glucosamine 6-phosphate. This substrate is required for the biosynthesis of one of the HA capsule monomers UDP-GlcNAc, which is also essential for peptidoglycan and lipopolysaccharide biosynthesis. Also identified was the gene *pgl*, which encodes a protein with a NagB domain. In E. coli, NagB is a 6-phosphogluconolactonase/glucosamine-6-phosphate deaminase that converts α-d-glucosamine 6-phosphate to β-d-fructose. GlmS acts at the same position in the pathway but in the reverse direction (39). Experiments in E. coli strain K-12 showed that *glmS* mutants were viable so long as glucosamine-6-phosphate or *N*-acetylglucosamine-6-phosphate (converted to glucosamine by *nagA*) was available in the growth media (40). However, more recent TraDIS experiments identified *glmS* as an essential gene when strain K-12 was grown *in vitro* in Luria broth (41). Multiple studies have shown that the gene *glmM*, encoding a phosphoglucosamine mutase, and *glmU*, encoding a fused *N*-acetylglucosamine-1-phosphate uridylyltransferase, which are required for the last two steps in the UDP-GlcNAc biosynthesis pathway, are essential for the viability of E. coli (41–43). Initial TraDIS analysis of the VP161-Tn*7 Himar1* library grown *in vitro* in rich media revealed that there were no VP161-Tn*7 Himar1* mutants with insertions in *glmM* or *glmU* (Table S3), indicating that these genes are also essential in P. multocida.

The genes encoding RelA and SpoT, proteins involved in regulation of the stringent response, were both identified as important for capsule production. The stringent response is a bacterial stress response induced by nutrient starvation and is activated by increased concentration of the nucleotide alarmones (p)ppGpp (44, 45). The concentration of (p)ppGpp is controlled by RSH proteins together with small alarmone synthase (SAS) and hydrolase (SAH) proteins (24, 44). When nutrient availability is low, RSH, SAS, and SAH proteins respond by increasing (p)ppGpp synthase activity and/or decreasing (p)ppGpp hydrolase activity (46–48). The (p)ppGpp alarmone molecules activate the stringent response by binding to several protein targets and allosterically regulating their activity (49, 50). Targets of (p)ppGpp vary depending on the RSH enzyme and bacterial species but include proteins involved in DNA replication, translation, and nucleotide biosynthesis (51–54). The result is a halt in cell growth and the induction of amino acid biosynthesis and other stress responses to alleviate the nutrient limitation. In addition, (p)ppGpp can bind to RNA polymerase (RNAP) alone or with the RNAP-binding transcription factor DskA (55, 56), changing promoter affinity of RNAP that results in genome-wide changes in gene expression (57, 58). In other bacterial species, the stringent response positively regulates several virulence factors, including capsule production in Staphylococcus aureus (44, 59, 60). The two RSH proteins RelA and SpoT produced by P. multocida share a high level of identity with the equivalent proteins in other bacterial species, including the C-terminal regulatory region containing the TGS, helical, CC/ZFD, and ACT domains that have been shown in other bacterial species to autoregulate the (p)ppGpp synthase/hydrolase activity (61). No other RSH, SAS, or SAH proteins are present in the VP161 genome.

The TraDIS essential gene data indicated that insertions into the 5′ region encoding the (p)ppGpp hydrolase and synthase domains of *spoT* were lethal, and TraDISort data indicated that insertions in the 3′ regulatory region were associated with a high-density cell phenotype and an acapsular colony morphology. Further experiments showed that 3′ *spoT* mutants had reduced expression of *hyaD*, encoding the glycosyltransferase essential for HA capsule biosynthesis, and reduced amounts of HA capsule. We predict that mutants with insertions in the 3′ region of *spoT* produce functional truncated proteins that have lost autoregulatory function and have uncontrolled (p)ppGpp synthase activity leading to an increase in cellular levels of (p)ppGpp. Interestingly, the expression of the gene encoding the global regulator Fis is negatively regulated by the stringent response in E. coli (30, 62). If the P. multocida Fis were also negatively regulated by the stringent response, an increase in (p)ppGpp concentration would result in reduced *fis* expression in this species. However, our experiments showed that the expression of *fis* was unchanged in the P. multocida 3′ *spoT* mutant, indicating that the activation of the stringent response results in reduced expression of capsule genes in a Fis-independent manner. Other possible mechanisms of reduced capsule gene expression include reduced affinity of RNAP for capsule biosynthesis gene promoters or the *hfq* promoter when bound to (p)ppGpp. Alternatively, (p)ppGpp may allosterically regulate the activity of Hfq. Furthermore, as Hfq is an RNA chaperone known to interact with small RNAs to regulate gene translation (15), there may be Hfq-dependent small RNAs that regulate capsule biosynthesis gene expression in P. multocida. However, the density of the P. multocida VP161-Tn*7 Himar1* insertions in our library was insufficient to identify any such small RNA genes (typically <250 nucleotides [nt] in length).

TraDIS data analysis of the VP161-Tn*7 Himar1* mutant library following growth in rich media identified *Himar1* insertions throughout the *relA* gene; however, following TraDISort analysis of the LD2 and HD2 cell layers, insertions in *relA* were identified only in the 3′ regulatory region of the gene. This suggested that *relA* mutants with insertions in the 5′ region of the gene, which would have disrupted (p)ppGpp synthase activity, were fully capsulated and partitioned only to the upper cell layer following the first gradient centrifugation. Moreover, the majority of the 3′ *relA* mutants recovered from the second gradient separated into the bottom, HD2 cell layer following centrifugation, correlating with an acapsular phenotype. In E. coli, removing the 3′ regulatory region of *relA* or disrupting the ACT domain results in truncated proteins that have increased (p)ppGpp synthesis activity (25, 28). We predict that 3′ *relA* mutants have increased (p)ppGpp synthesis due to the loss of the autoregulatory ACT domain. As above, we predict this increase in (p)ppGpp results in altered protein activity or regulation of genes required for HA capsule production. Attempts were made to construct a directed TargeTron mutant in the 3′ end of *relA*, but these were unsuccessful despite multiple attempts.

Several other genes identified as important for capsule production in P. multocida were also associated with activation of the stringent response, including *ptsH*, *ppx*, *tufA_1*, and *tufA_2*. The gene *ptsH* encodes HPr, a component of the phosphoenolpyruvate:sugar phosphotransferase (PTS) system that imports and phosphorylates carbohydrate monomers. No other PTS system genes were identified as important for capsule production by the TraDISort analysis, suggesting that import and phosphorylation of carbohydrate monomers by the PTS system are not required for HA production in strain VP161. In E. coli, HPr indirectly regulates SpoT activity by binding to phosphorylated Rsd during carbon downshift, stopping Rsd from binding to SpoT and inducing (p)ppGpp synthesis (63). In Caulobacter cresentus during glutamine starvation, HPr is required for phosphorylating EIIA^Ntr^, which then binds to SpoT (64). HPr in P. multocida may have a similar role, binding to or controlling the phosphorylation state of proteins that regulate the activity of SpoT and/or RelA. The *ppx* gene is also predicted to be involved in the stringent response, as it encodes an exopolyphosphatase that cleaves terminal phosphates from long-chain polyphosphate (polyP) (65). Phosphate starvation in E. coli results in an increase in (p)ppGpp concentration due to altered SpoT activity (66). Disrupting *ppx* likely reduces available phosphate within the cell and increases (p)ppGpp synthesis and/or decreases (p)ppGpp hydrolysis by SpoT. The genes *tufA_1* and *tufA_2* both encode elongation factor Tu (EF-Tu), which delivers charged tRNAs to the ribosome by forming a complex with the tRNA and GTP (67). The EF-Tu-tRNA-GTP complex competes with RelA bound to uncharged tRNAs for binding to the ribosome A site. Reduced EF-Tu levels from inactivation of either *tufA_1* or *tufA_2* may result in an increased number of RelA-uncharged tRNA complexes interacting with the ribosome, and this would result in increased (p)ppGpp synthesis.

In addition to *tufA_1* and *tufA_2*, other genes encoding translation factors were identified by TraDISort as important for HA capsule biosynthesis in P. multocida strain VP161, including *efp*, *empA*, and *empB*. The gene *efp* encodes elongation factor P (EF-P), which restores ribosomes stalled during translation of polyproline stretches (68, 69). The genes *empA* and *empB* encode an EF-P lysine lysyltransferase that adds R-β-lysine to Lys34 within EF-P and a lysine 2,3-aminomutase that converts l-lysine to R-β-lysine, respectively (70, 71). Both *empA* and *empB* are required for correct EF-P function in E. coli (70). Mutation of *efp*, *empA*, or *empB* in E. coli leads to reduced production of several capsule-associated genes, some of which are homologs of genes identified as important for P. multocida capsule production by TraDISort analysis, including Rnb and several ATP synthase subunit proteins (72). Therefore, EF-P is likely important for capsule production in P. multocida, as it facilitates the production of other proteins crucial for HA capsule production.

Overall, we have identified genes required for growth in rich media and comprehensively characterized HA capsule production in P. multocida strain VP161. To our knowledge, this is the first time a whole-genome saturation mutagenesis approach has been used to identify essential genes and those associated with the production of extracellular capsule in P. multocida, an important virulence factor for this pathogen. Importantly, the stringent response was shown to be a negative regulator of capsule biosynthesis genes. The RSH proteins, SpoT and RelA, critical for the synthesis and control of the levels of the alarmone nucleotides (p)ppGpp and the associated regulatory proteins, TufA_1, TufA_2, HPr, and PPX, were all identified by TraDISort as important for HA capsule production. Further work is required to elucidate the exact mechanism by which these stringent response proteins precisely control capsule production in this pathogen.

## MATERIALS AND METHODS

### Bacterial strains, plasmids, and culturing conditions.

Bacterial strains and plasmids used in this study are listed in Table S1 in the supplemental material. P. multocida strains were cultured in HI broth (Oxoid). E. coli strains were cultured in lysogeny broth (LB). P. multocida and E. coli were grown at 37°C, with liquid broth cultures shaken at 200 RPM. Solid media were produced by adding 1.5% (wt/vol) agar to the media. When required, media were supplemented with appropriate antibiotics at the following concentrations: kanamycin (50 μg/mL), spectinomycin (50 μg/mL), and tetracycline (2.5 μg/mL).

### DNA manipulations, Sanger sequencing, and oligonucleotides.

Genomic DNA was extracted using the HiYield genomic DNA minikit (RBC bioscience) and plasmid DNA was extracted using the NucleoSpin plasmid kit (Macherey-Nagel), as per the manufacturer’s instructions. DNA samples were quantified using a NanoDrop 1000 Spectrophotometer or Qubit fluorometer (Thermo Fisher Scientific). PCRs were performed using *Taq* DNA polymerase (Roche), Phusion high-fidelity DNA polymerase (NEB), or KAPA HiFi HotStart DNA polymerase (Roche) as per the manufacturer’s instructions. Restriction digestion and ligation reactions were performed using enzymes from Roche or New England Biolabs, as per the manufacturer’s instructions. Oligonucleotides used in this study were synthesized by Sigma-Aldrich and are listed in Table S4 in the supplemental material. Sanger sequencing reactions were performed using plasmid or genomic DNA as the template with BigDye Terminatior version 3.1 (Applied biosystems) as described previously (73). Sanger sequencing data were analyzed using Geneious Prime (Biomatters).

### Transformation and conjugation.

Transformation of P. multocida was performed as described previously (14), with the electroporation conditions modified to 2 kV, 600 Ω, and 25 μF. Conjugation experiments were performed by filter mating with E. coli donor and P. multocida recipient VP161-Tn7 (kanamycin-resistant parent, see supplemental material for construction details) as follows: cultures were each grown to mid-exponential stage (OD_600_ of ∼0.5) and washed in 1× phosphate-buffered saline (PBS), and then 1 mL of each donor and recipient culture was mixed and filtered through a 0.22-μm nylon filter using a syringe and Swinnex filter holder. The filter containing the cells was then aseptically placed (cell-side up) onto an HI plate without antibiotics and incubated at 37°C for 1 h. Following incubation, we resuspended the cells in 1 mL of HI by vortexing the filter and then plated them onto HI agar plates containing spectinomycin, kanamycin, or spectinomycin and kanamycin to select for growth of donor, recipient, or transconjugants, respectively. Following overnight incubation at 37°C, viable counts were performed to determine CFU/mL of donor, recipient, and transconjugants.

### Site-directed mutagenesis of selected genes using TargeTron.

Site-directed mutagenesis of specific genes in the VP161 genome was performed using the P. multocida TargeTron plasmid pAL953 retargeted to the appropriate gene using the intron site finder Clostron (74) as described previously (16, 73). A full list of retarget primers is shown in Table S4. TargeTron intron insertion into the target gene was confirmed by PCR amplification of the target region using two PCRs. The first reaction used primers flanking the gene of interest and the second used the TargeTron-specific primer paired with the appropriate primer flanking the target gene (Table S4). Direct Sanger sequencing was also conducted using genomic DNA as the template with the TargeTron-specific outward-firing primer BAP6544 to confirm that only a single intron insertion was present in the genome at the specific target site.

### Construction of complementation vectors.

We complemented selected P. multocida mutants by providing a wild-type copy of the target gene on the P. multocida expression plasmid pAL99S, which conferred spectinomycin resistance and was suitable for the TargeTron mutants, or pAL99T, which conferred tetracycline resistance and was suitable for the *Himar1* mutants (Table S1). To produce each of the complementation vectors, we PCR-amplified the target gene using Phusion polymerase with P. multocida VP161 genomic DNA as the template, a forward primer containing an appropriate restriction site followed by a ribosome binding site, and a reverse primer with an appropriate restriction site (Table S4). Purified PCR products were digested with the appropriate restriction enzymes and ligated into similarly digested pAL99S or pAL99T. Sanger sequencing was used to confirm that each plasmid complementation vector contained a wild-type copy of the target gene. To produce each complementation strain, we used the appropriate plasmid (Table S1) to transform the appropriate P. multocida mutant strain by electroporation. To produce mutant strains harboring empty vector (control strains), pAL99S or pAL99T was used to separately transform each mutant (Table S1).

### Generation of the P. multocida *Himar1* transposon mutant library and growth in rich media.

P. multocida transposon mutant libraries were produced by delivery of the *Himar1* transposon-containing suicide vector pAL614 into P. multocida by conjugation between donor E. coli strain AL2972 and P. multocida strain VP161-Tn*7* (Table S1). Following overnight growth, *Himar1* transconjugants were scraped from HI agar plates supplemented with both kanamycin and spectinomycin, pooled in 15% glycerol broth, and then stored at −80°C in 1-mL aliquots containing ∼6.45 × 10^6^ VP161-Tn*7 Himar1* mutants. For identification of genes essential for growth in rich media, two aliquots of the VP161-Tn*7 Himar1* mutant library (named A and B) were grown overnight in 50 mL of HI broth, subcultured into 50 mL of fresh HI broth, and grown until mid-exponential growth phase (OD_600_ of ∼0.5). Two 1-mL aliquots of surviving mutants were recovered from each mutant library culture (A1, A2, B1, B2) and used for TraDIS analysis.

### Discontinuous Percoll gradient centrifugation.

Separation of capsular and acapsular mutant strains in the VP161-Tn*7 Himar1* mutant library was performed using Percoll density gradient centrifugation as described previously (17, 75) with the following modifications. For each discontinuous gradient, an 80% Percoll (in PBS wt/vol) bottom layer, a 40% Percoll middle layer, and a 10% Percoll top layer were added to a polyallomer centrifuge tube (17 mL, 16 mm by 102 mm, Beckman). Cells were grown to mid-exponential-phase growth, and 20 mL of each culture was centrifuged, resuspended in 1 mL of PBS, and gently loaded onto the top of each gradient. Gradients were then centrifuged in a fixed angle rotor for 30 min at 3,000 × *g*. Cell layers of interest were recovered by syringe aspiration. To remove excess Percoll, the cell pellet was washed with 1 mL of PBS, centrifuged, and resuspended in 1 mL of PBS. Three replicates of the *Himar1* library were subjected to discontinuous gradient centrifugation as described above. Following the first centrifugation, the top layer of cells consisting of the capsulated population of the mutant library was not examined further. Cells recovered from the bottom cell layer were grown to mid-exponential growth stage for 4 h in HI broth (37°C with shaking at 200 rpm) and subjected to a second discontinuous Percoll gradient centrifugation using the same conditions as described above. The top (LD2) cell layer and the bottom (HD2) cell layer generated by the second gradient centrifugation were collected separately and used for TraDISort analysis.

### TraDIS library production, sequencing, and data analysis.

TraDIS libraries for Illumina sequencing were produced as described previously (76) with minor modifications. A summary (Fig. S1) and full details of TraDIS library production, Illumina MiSeq sequencing, and data analysis methods are provided in the supplemental material.

TraDIS libraries were submitted to the Micromon Genomics facility at Monash University and sequenced on an Illumina MiSeq v2 using 150-bp single-end sequencing with the custom *Himar1* sequencing oligonucleotide BAP8042 (Table S4). Poor-quality reads and Illumina adapter sequences were removed from the sequencing data using Trimmomatic v0.38. The sequencing reads were then analyzed using Bio-TraDIS v1.4.1 and modified scripts as described previously (17, 76). All reads beginning with a correct *Himar1* transposon tag (5′-CAACCTGT-3′) were aligned to the reference P. multocida VP161 genome sequence, with the first base of each alignment deemed to be a transposon insertion site (VP161 NCBI database accession no. CP048792.1). Genes essential for growth *in vitro* in HI were defined as those with an insertion index (number of unique insertions relative to gene length) of less than 0.0134, with data from all rich media TraDIS libraries (A1, A2, B1, B2) combined for analysis. Specific metabolic pathways for each of the P. multocida genes predicted to be essential were identified by comparison to the Kyoto Encylopedia of Genes and Genomes (KEGG) database by BlastKOALA (77) and to the UniProt database by BLASTp (78). Genes associated with high-density/reduced capsule production were identified by comparing normalized read counts for each gene in TraDIS libraries generated from the upper, low-cell-density layer (LD2 cell layer) with TraDIS libraries generated from the lower, high-density cell layer (HD2 cell layer). Any gene with a significant increase in the number of *Himar1* insertion sites identified in mutants recovered from the HD2 cell layer (log_2_ > 2.0, *q* value of <0.001, insertion index ratio of >0.8) compared to that in mutants recovered from the LD2 cell layer was identified as important for capsule production. Homologs of genes identified by TraDISort were identified by comparison to the Kyoto Encyclopedia of Genes and Genomes (KEGG) database by BlastKOALA (77) and to the UniProt database by BLASTp (78).

### Measurement of hyaluronic acid capsule production.

Capsular polysaccharide was extracted from P. multocida cells grown in broth and measured as described previously (14) with minor modifications. Briefly, cells were harvested from 1 mL of mid-exponential culture using centrifugation at 13,000 × *g* for 10 min, washed with PBS, and resuspended in 1 mL of PBS. The cells were then heated at 42°C for 1 h and pelleted by centrifugation at 13,000 × *g* for 10 min, and the supernatant containing capsular polysaccharide was recovered. For each sample, 100 μL of supernatant was mixed with 900 μL of stains-all solution (14) and the absorbance at 640 nm was measured. The absorbance of each sample was compared to hyaluronic acid standards (between 2.5 μg/mL and 40 μg/mL) to determine the concentration of hyaluronic acid. Mann-Whitney U tests were performed to determine if differences in the amount of hyaluronic acid produced by different strains were significant.

### Scanning electron microscopy.

SEM was performed on P. multocida strains grown to mid-exponential growth phase (OD_600_ of 0.5 to 0.6). Cells were filtered onto a 0.4-μm aperture polycarbonate membrane and prepared for imaging using lysine acetate-ruthenium red-osmium fixation, critical point drying, and sputter coating as previously described with minor modifications (79). For the fixation solution, 100 μL of 16% formaldehyde was used in place of 80 μL of 25% formaldehyde solution. Cells were imaged using a field emission scanning electron microscope Nova NanoSEM 450 with an accelerating voltage of 5 kV in secondary electron mode with a working distance of ∼4.5 mm. Images were taken under immersion mode through the lens detector. Cell widths were measured from electron micrographs generated by SEM using the FIJI package for ImageJ. Significant differences in cell widths were determined using an unpaired *t* test.

### RNA extraction and qRT-PCR.

RNA was isolated from P. multocida cells grown to mid-exponential-phase growth (OD_600_ of ∼0.4) in biological quadruplicate using TRIzol reagent (ThermoFisher Scientific) as per the manufacturer’s instructions. RNA samples were DNase treated using the RNeasy kit (Qiagen) as per the manufacturer’s instructions, and then RNA was purified using phenol:choloroform extraction with 5 PRIME phase lock gel tubes (Quanta Biosciences) following the manufacturer’s instructions followed by sodium acetate-ethanol precipitation. cDNA was synthesized for qRT-PCR using the AffinityScript cDNA synthesis kit (Agilent Technologies) as per the manufacturer’s instructions. Each sample was used in cDNA synthesis reactions with (+RT) or without (−RT) reverse transcriptase. The qRT-PCRs were performed using Brilliant II SYBR green qPCR master mix (Agilent Technologies) per the manufacturer’s instructions with primers specific to P. multocida strain VP161 *gyrB*, *hyaD*, or *fis* (Table S4), with reactions performed in an Aria Mx real-time PCR system (Agilent). Biological quadruplicate cDNA samples were each measured in technical duplicate. The −RT samples were analyzed to ensure no products were amplified before 35 cycles, and the melt curves from +RT reactions were analyzed to ensure only a single product was amplified in each reaction. Significant differences in gene expression were determined using a Mann-Whitney U test.

### Data analysis.

Sequencing data and analysis output files have been uploaded to the NCBI Gene Expression Omnibus database under accession number GSE198087.
