# Supplementary material for: Genome-Wide Investigation of Pasteurella multocida Identifies the Stringent Response as a Negative Regulator of Hyaluronic Acid Capsule Production
Source: Microbiol Spectr. 2022 Apr 11;10(2):e00195-22. doi: 10.1128/spectrum.00195-22 (PMC9045168; doi:10.1128/spectrum.00195-22)
Supplement: SUPPLEMENTAL FILE 1 — Supplemental material. Download spectrum.00195-22-s0001.pdf, PDF file, 1.2 MB [file spectrum.00195-22-s0001.pdf]

Supplementary material

Genome-wide investigation of *Pasteurella multocida* identifies the stringent response as a negative regulator of hyaluronic acid capsule production

Construction of a *P. multocida* strain VP161 Tn*7* mutant

To allow for selection of *Himar*1 transposants following conjugation, the fowl cholera isolate VP161 (1) (Table S1) was modified by the introduction of the transposon Tn*7*, encoding a kanamycin resistance gene, *aph(3')*. Triparental mating conjugation was performed with *P.* multocida strain VP161, *E. coli* strain AL2017, containing the donor plasmid pAL1043, and *E. coli* strain AL2621, containing the helper plasmid, pTNS3 (2) (Table S1). Tn*7* inserts into a single conserved intergenic region approximately 23 bp downstream of *glmS* and has been shown not to disrupt virulence in a wide range of bacterial species (7). A single Tn*7* insertion at the expected site in strain VP161 was confirmed by direct Sanger sequencing of VP161-Tn*7* genomic DNA using the Tn*7*-specific primer BAP8265 (Table S3).

Detailed TraDIS library production method

The TraDIS library production method used in this study was adapted from a previous study (3), for use with a large *P. multocida* strain VP161-Tn*7 Himar1* mutant library. VP161-Tn*7 Himar1* mutants were recovered following growth in rich media, or were recovered from the LD2 and HD2 cell layers following consecutive Percoll gradient centrifugations. Genomic DNA was extracted from the recovered mutants using the HiYield genomic DNA MiniKit (RBC bioscience).

A total of 6 µg of genomic DNA in 50 µL of dH_2O was sheared to ~300 bp fragments using Covaris Adaptive Focused Acoustics (AFA) with the following settings: Duty Cycle – 20%, Intensity – 5, Cycles – 200. Samples were sheared in Covaris microTUBEs with AFA Fibre and Snap-Caps. The sheared DNA fragments were purified using AxyPrep Mag PCR Purification Kit (Axygen) magnetic beads as per manufacturer's instructions with the exception that 80% EtOH was used instead of

70% EtOH. Sheared DNA samples were purified using a 1.5 x volume of magnetic beads with eluted in 51 µL dH_2O. The Sheared DNA fragments were then end-repaired using NEBNext End Repair module (NEB) as per the manufacturer's instructions using 50 µL of the purified fragmented DNA. The end-repaired DNA fragments were then purified using a 1.5x volume of magnetic beads with elution in 32 µL of dH_2O. The full aliquot of purified, end-repaired fragments was then 3' A-tailed using the NEBNext dA-tailing module (NEB) as per the manufacturer's instructions. The A-tailed fragments were then purified using a 1.8 x volume of magnetic beads with elution in 20 µL of dH_2O. Splinkerette adapters were then ligated to the ends of purified 3' A-tailed DNA fragments. The top and bottom strand splinkerette oligonucleotides (BAP8034 and BAP8035 in Table S3) were annealed by mixing at a final concentration of 50 µM for each oligonucleotide and incubating at 99°C for 15 minutes. The annealed splinkerette adapter was allowed to cool at room temperature then ligated onto the A-tailed DNA fragments using the NEBNext Quick Ligation Module (NEB), as per the manufacturer's instructions using 1 µL of the annealed adapter. The adapter-ligated fragments were then purified using a 1 x volume of magnetic beads with elution in 50 µL of dH_2O, followed by a 0.8 x volume of magnetic beads with elution in 20 µL of dH_2O

The purified, adapter-ligated, DNA fragments were then used as template for PCR amplification to produce TraDIS libraries using KAPA HiFi HotStart ReadyMix PCR Kit (Roche). Each PCR was setup as follows: 25 µL of 2 x KapaHiFi HotStart ReadyMix, 1 µM each of the P5 *Himar1*-specific and P7 Adapter-specific primers (Table S3), either 100 ng or 250 ng of purified adapter-ligated DNA and the volume adjusted with sterile water to give a final volume of 50 µL. Rich media TraDIS libraries were generated with either 100 ng (rich media libraries A1 and B1) or 250 ng of DNA as template (rich media libraries A2 and B2). All TraDISort libraries were generated using

250 ng of template. TraDIS library amplifications were performed using the following thermocycler amplification conditions: 95°C for 2 min, 19 x [98°C for 20 sec, 65°C for 30 sec, 72°C for 45 sec], 72°C for 2 min, 12°C hold. All amplified TraDIS libraries were then purified using a 0.8 x volume of magnetic beads with elution in 32 µL of dH_2O.

Following the shearing, adapter ligation and library PCR amplification steps, 1 µL of the purified DNA was diluted 1:10 in dH_2O and used to determine DNA concentration and size range using Qubit fluorometric quantification (Thermo Fisher) and Fragment Analyzer (Agilent) analyses. DNA size ranging was used to confirm Covaris shearing produced DNA fragments of ~300 bp and that there was minimal adapter dimer produced during the adapter ligation step. Next, the amount of Illumina flow cell clusterable DNA was determined using the KAPA Library Quantification Kit KK4824 (Roche) as per the manufacturer's instructions (universal qPCR Master Mix setup with no ROX). The TraDIS libraries were diluted 1:10,000 and 1:100,000 for this qPCR analysis. Following the above quality controls, all libraries were sequenced on an Illumina MiSeq v2 using 150 bp single end sequencing using the custom *Himar1*-specific oligonucleotides BAP8042 and BAP8043 that were used to sequence *Himar1* transposon-chromosome junctions and index sequences in the splinkerette adapter, respectively.

The TraDIS illumina sequencing data was trimmed to remove adapter sequences and poor-quality data using Trimmomatic v0.38 (9), using all adapter-specific primers and Illumina P5 and P7 sequences. Trimmomatic was run with the following settings: leading:3, trailing:3, slidingwindow:4:15, minlength:1, crop:40. The TraDIS sequencing data was then analysed using Bio-TraDIS v1.4.1 and modified scripts available at https://github.com/sanger-pathogens/Bio-Tradis and https://github.com/francesca-short/tradis_scripts (8, 10). *Himar1* insertion sites were mapped to the VP161

reference genome using the bacteria_tradis script with the following settings: --smalt –smalt_r 0 -m 0 -t CCAACCTGT. The correlation of the number of unique *Himar1* insertion sites per gene between TraDIS library replicates was high (Fig. S2 and Fig. S5), allowing data from library replicates to be combined using combine_tradis_plots. Principal component analysis (PCA) was also performed to show reproducibility of the TraDIS library replicates. PCA analysis was performed using the prcomp function using R, and plots were generated using the packages ggbiplot, ggplot2, ggrepel and reshape2. For determining rich media essential genes, tradis_gene_insert_sites was run with -trim3 0.1 to remove insertion sites from the final 10% at the 3' end of the gene. Genes important for capsule production in *P. multocida* were determined using the scripts tradis_gene_insert_sites run with no trimming, and TraDIS_comparison_positive_selection.R with the settings -q 0.001 -i 0.8, with LD2 TraDIS libraries as the control and HD2 libraries as the condition.

References

1. **Wilkie IW, Grimes SE, O'Boyle D, Frost AJ.** 2000. The virulence and protective efficacy for chickens of *Pasteurella multocida* administered by different routes. *Vet Microbiol* **72:**57-68.
2. **Choi KH, Gaynor JB, White KG, Lopez C, Bosio CM, Karkhoff-Schweizer RR, Schweizer HP.** 2005. A Tn7-based broad-range bacterial cloning and expression system. *Nat Methods* **2:**443-8.
3. **Barquist L, Mayho M, Cummins C, Cain AK, Boinett CJ, Page AJ, Langridge GC, Quail MA, Keane JA, Parkhill J.** 2016. The TraDIS toolkit: sequencing and analysis for dense transposon mutant libraries. *Bioinformatics* **32:**1109-11.
4. **Chung JY, Wilkie I, Boyce JD, Townsend KM, Frost AJ, Ghoddusi M, Adler B.** 2001. Role of capsule in the pathogenesis of fowl cholera caused by *Pasteurella multocida* serogroup A. *Infect Immun* **69:**2487-92.
5. **Harper M, St Michael F, John M, Vinogradov E, Steen JA, van Dorsten L, Steen JA, Turni C, Blackall PJ, Adler B, Cox AD, Boyce JD.** 2013. *Pasteurella multocida* Heddleston serovar 3 and 4 strains share a common lipopolysaccharide biosynthesis locus but display both inter- and intrastrain lipopolysaccharide heterogeneity. *J Bacteriol* **195:**4854-64.
6. **Simon R, Priefer U, Pühler A.** 1983. A broad host range mobilization system for in vivo genetic engineering: transposon mutagenesis in gram negative bacteria. *Nat Biotechnol* **1:**784-791.
7. **Murray GL, Morel V, Cerqueira GM, Croda J, Srikram A, Henry R, Ko AI, Dellagostin OA, Bulach DM, Sermswan RW, Adler B, Picardeau M.** 2009.

Genome-wide transposon mutagenesis in pathogenic *Leptospira* species. *Infect Immun* **77:**810-6.

Supplementary Tables and Figures

Table S1. Strains and plasmids used in this study.

Strain or plasmid	Description	Source or reference
**Strains**		
P. multocida		
VP161	Avian isolate strain, serotype A:1	(1)
VP161-Tn*7*	VP161 with Tn*7* insertion downstream of *glmS;* Kan^R	This study
PBA930	Serotype A:1 strain X73 *hexA*::*tet*(M) mutant, Tet^R	(4)
AL2188	VP161 harbouring pAL99S; Spec^R	(5)
AL2232	VP161 harbouring pAL99T; Tet^R	(5)
AL3544	VP161 *ppx* TargeTron mutant, intron inserted between nucleotides 474-475; Kan^R	This study
AL3574	VP161 *hyaD* TargeTron mutant, intron inserted between nucleotides 1483-1484; Kan^R	This study
AL3684	AL3145 harbouring pAL99T; Tet^R	This study
AL3685	VP161 *hexA* TargeTron mutant, intron inserted between nucleotides 123-124; Kan^R	This study
AL3793	VP161 *ptsH* TargeTron mutant, intron inserted between nucleotides 58-59; Kan^R	This study
AL3818	VP161-Tn*7* *pgm Himar1* mutant, transposon inserted between nucleotides 121-122; Spec^R	This study
AL3819	VP161-Tn*7* *galU Himar1* mutant, transposon inserted between nucleotides 476-477; Spec^R	This study
AL3874	AL3818 harbouring vector pAL99T; Spec^R Tet^R	This study
AL3876	AL3818 harbouring pAL1676; Spec^R Tet^R	This study
AL3878	AL3819 harbouring vector pAL99T; Spec^R Tet^R	This study
AL3880	AL3818 harbouring pAL1678; Spec^R Tet^R	This study
AL3953	AL3793 harbouring vector pAL99S; Kan^R Spec^R	This study
AL3954	AL3793 harbouring pAL1687; Kan^R Spec^R	This study
AL3955	AL3544 harbouring vector pAL99S; Kan^R Spec^R	This study
AL3956	AL3544 harbouring pAL1690; Kan^R Spec^R	This study
AL3957	AL3685 harbouring vector pAL99S; Kan^R Spec^R	This study
AL3958	AL3685 harbouring pAL1691; Kan^R Spec^R	This study
AL3961	AL3574 harbouring vector pAL99S; Spec^R	This study
AL3962	AL3574 harbouring pAL1693; Spec^R	This study
AL3991	VP161 *spoT* TargeTron mutant, intron inserted between nucleotides 1779-1780; Kan^R	This study
AL3993	AL3991 harbouring vector pAL99S; Kan^R Spec^R	This study
AL3994	AL3991 harbouring pAL1700; Kan^R Spec^R	This study
E. coli		
DH5α	F$^-$ deoR endA1 gyrA96 hsdR17 (r_K^- m_K^-) recA1 relA1 supE44 thi-1 Δ(lacZYAargFV169) ϕ80lacZΔM15	Bethesda Research Laboratories
S17-1 λ*pir*	(F$^-$) RP4-2-Tc::Mu *aphA*::Tn*7recA*λ*pir* lysogen Sm^r Tp^r	(6)
AL1296	DH5α harbouring pAL99S; Spec^R	(5)
AL1995	DH5α harbouring pAL953; Kan^R Spec^R	(5)
AL2017	S17-1 λ*pir* harbouring pTNS3; donor strain for Tn*7* mutagenesis	Boyce Laboratory
AL2224	DH5α harbouring pAL99T; Tet^R	(5)
AL2621	S17-1 λ*pir* harbouring pAL1083; donor strain for Tn*7* mutagenesis	Boyce Laboratory
AL2972	S17λpir harbouring pAL614, Amp^R Spec^R	This study

Strain or plasmid	Description	Source or reference
**Plasmids**		
pTNS3	Tn*7* helper plasmid encoding the Tn*7* site-specific transposition pathway *tnsABCD*; Amp^R	(2)
pAL1083	Tn*7* delivery vector; mini-Tn*7* containing Kan^R oriColE1 Amp^R	Boyce Laboratory
pAL99S	*P. multocida-E. coli* expression plasmid; $Spec^R$ P*tpiA*	(5)
pAL99T	*P. multocida-E. coli* expression plasmid; Tet^R P*tpiA*	(5)
pAL614	RP4 mobilisable plasmid containing *Himar1*::Spec; RP4 Mob^+ oriR6K Amp^R $Spec^R$ tnpC9	(7)
pAL953	*P. multocida* vector containing TargeTron group II intron; $Spec^R$ Kan^R	(5)
pAL1337	pAL953 with group II intron targeted to *hyaD*; $Spec^R$	Boyce Laboratory
pAL1504	pAL953 with group II intron targeted to *hexA*; $Spec^R$ Kan^R	This study
pAL1511	pAL953 with group II intron targeted to *ppx*; $Spec^R$ Kan^R	This study
pAL1600	pAL953 with group II intron targeted to *ptsH*; $Spec^R$ Kan^R	This study
pAL1658	pAL953 with group II intron targeted to *spoT*; $Spec^R$ Kan^R	This study
pAL1676	*pgm* from VP161 cloned into pAL99T, Tet^R	This study
pAL1678	*galU* from VP161 cloned into pAL99T, Tet^R	This study
pAL1687	*ptsH* from VP161 cloned into pAL99S, $Spec^R$	This study
pAL1690	*ppx* from VP161 cloned into pAL99S, $Spec^R$	This study
pAL1691	*hexA* from VP161 cloned into pAL99S, $Spec^R$	This study
pAL1693	*hyaD* from VP161 cloned into pAL99S, $Spec^R$	This study
pAL1700	*spoT* from VP161 cloned into pAL99S, $Spec^R$	This study

Table S2. Rich media, high density layer 2 and low-density layer 2 TraDIS library sequencing data statistics.

Library	Number of reads	Reads with a Tn tag	Mapped reads	Number of unique insertion sites
Rich media				
A1	1,422,287	1,289,050	908,523	40,878
A2	2,155,825	1,954,820	1,371,448	47,077
B1	1,727,185	1,566,734	1,034,180	42,551
B2	2,635,359	2,397,821	1,703,987	51,336
Total	7,940,656	7,208,425	5,018,138	81,292
Low density layers				
LD2 A	1,710,725	1,606,889	1,262,387	33,666
LD2 B	1,080,308	1,015,170	798,456	28,640
LD2 C	691,291	649,925	512,738	25,723
Total	3,482,324	3,271,984	2,573,581	42,878
High density layers				
HD2 A	771,313	723,731	554,705	11,812
HD2 B	475,378	446,985	350,625	6,665
HD2 C	751,843	698,363	532,265	10,685
Total	1,998,534	1,869,079	1,437,595	19,487

Table S3. List of *P. multocida* strain VP161 genes essential for growth in rich media. Essential genes are defined as those with an insertion index (number of unique insertions relative to gene length) of less than 0.0134.

VP161 Locus tag	Gene name	Gene length	Unique *Himar1* insertions	Total *Himar1* insertions	Insertion index	Function	COGs group[a]	Homolog in DEG
PmVP161_0001	*dnaA*	1356	7	15	0.005733	Chromosomal replication initiator protein DnaA	L	+
PmVP161_0002	*dnaN*	1101	2	3	0.002018	Beta sliding clamp	L	+
PmVP161_0008	*dut*	456	0	0	0	Deoxyuridine 5'-triphosphate nucleotidohydrolase	F	+
PmVP161_0009	*coaBC*	1203	5	7	0.004617	Coenzyme A biosynthesis bifunctional protein CoaBC	H	+
PmVP161_0010	PmVP161_0010	675	8	10	0.013158	hypothetical protein	E	+
PmVP161_0011	*rpmB*	237	0	0	0	50S ribosomal protein L28	J	+
PmVP161_0012	*rpmG*	171	2	2	0.012987	50S ribosomal protein L33	J	+
PmVP161_0024	*rpmE*	213	2	2	0.010417	50S ribosomal protein L31	J	+
PmVP161_0025	*priA*	2268	15	126	0.007346	Primosomal protein N'	L	+
PmVP161_0028	*mdtA*	1212	5	7	0.004583	Multidrug resistance protein MdtA	M	+
PmVP161_0029	*msrC*	444	4	47	0.01	Free methionine-R-sulfoxide reductase	T	+
PmVP161_0030	*bepE*	3099	9	11	0.003226	Efflux pump membrane transporter BepE	V	+
PmVP161_0031	PmVP161_0031	1065	7	7	0.007299	hypothetical protein	R	+
PmVP161_0040	PmVP161_0040	76	0	0	0	tRNA-Val	S	-
PmVP161_0043	PmVP161_0043	76	0	0	0	tRNA-Val	S	-
PmVP161_0044	*gltX*	1443	8	10	0.006159	Glutamate--tRNA ligase	J	+
PmVP161_0045	PmVP161_0045	76	0	0	0	tRNA-Ala	S	-
PmVP161_0046	*pnp*	2145	11	30	0.005697	Polyribonucleotide nucleotidyltransferase	J	+
PmVP161_0053	*groL*	1644	4	5	0.002703	60 kDa chaperonin	O	+
PmVP161_0054	*groS*	291	0	0	0	10 kDa chaperonin	O	+
PmVP161_0058	*glyS*	2070	5	5	0.002684	Glycine--tRNA ligase beta subunit	J	+
PmVP161_0062	*glyQ*	903	4	5	0.00492	Glycine--tRNA ligase alpha subunit	J	+
PmVP161_0070	*accB*	462	2	2	0.004808	Biotin carboxyl carrier protein of acetyl-CoA carboxylase	I	+
PmVP161_0071	*accC*	1347	3	5	0.002473	Biotin carboxylase	I	+
PmVP161_0098	*polA*	2793	30	218	0.011933	DNA polymerase I	L	+
PmVP161_0104	*rep*	2016	13	14	0.007163	ATP-dependent DNA helicase Rep	L	+
PmVP161_0105	PmVP161_0105	93	0	0	0	tRNA-Ser	S	-
PmVP161_0106	PmVP161_0106	77	0	0	0	tRNA-Arg	S	-
PmVP161_0107	PmVP161_0107	77	0	0	0	tRNA-Arg	S	-
PmVP161_0115	*dapA*	897	4	5	0.00495	4-hydroxy-tetrahydrodipicolinate synthase	E	+
PmVP161_0121	*ybeY*	477	0	0	0	Endoribonuclease YbeY	J	+
PmVP161_0124	PmVP161_0124	192	1	1	0.00578	hypothetical protein	R	-
PmVP161_0135	*int*	1542	6	11	0.004323	Apolipoprotein N-acyltransferase	M	+
PmVP161_0136	*infA*	219	2	3	0.010101	Translation initiation factor IF-1	J	+

PmVP161_0140	*metK*	1155	6	7	0.005769	S-adenosylmethionine synthase	H	+
PmVP161_0145	*dapE*	1137	4	4	0.003906	Succinyl-diaminopimelate desuccinylase	E	+
PmVP161_0168	PmVP161_0168	1389	10	13	0.007994	hypothetical protein	H	+
PmVP161_0172	PmVP161_0172	87	0	0	0	tRNA-Leu	S	-
PmVP161_0173	PmVP161_0173	74	0	0	0	tRNA-Cys	S	-
PmVP161_0176	*trxA*	321	0	0	0	Thioredoxin 1	O	+
PmVP161_0177	*cobB*	705	6	438	0.009449	NAD-dependent protein deacylase	K	-
PmVP161_0179	*rne*	3018	11	64	0.004049	Ribonuclease E	J	+
PmVP161_0180	PmVP161_0180	90	0	0	0	tRNA-Ser	S	-
PmVP161_0189	*aspS*	1767	3	3	0.001886	Aspartate--tRNA ligase	J	+
PmVP161_0197	PmVP161_0197	321	1	2	0.00346	hypothetical protein	R	-
PmVP161_0198	*cydA*	1563	4	7	0.002843	Cytochrome bd-I ubiquinol oxidase subunit 1	C	+
PmVP161_0199	*cydB*	1137	2	2	0.001953	Cytochrome bd-I ubiquinol oxidase subunit 2	C	+
PmVP161_0205	PmVP161_0205	1155	5	15	0.004808	hypothetical protein	M	+
PmVP161_0209	PmVP161_0209	76	0	0	0	tRNA-Lys	S	-
PmVP161_0212	PmVP161_0212	76	0	0	0	tRNA-Lys	S	-
PmVP161_0230	PmVP161_0230	90	0	0	0	tRNA-Ser	S	-
PmVP161_0234	*cysS*	1380	6	8	0.004831	Cysteine--tRNA ligase	J	+
PmVP161_0252	PmVP161_0252	77	0	0	0	tRNA-Met	S	-
PmVP161_0255	*znuA*	1083	13	57	0.013333	High-affinity zinc uptake system protein ZnuA	P	+
PmVP161_0257	PmVP161_0257	132	1	1	0.008403	hypothetical protein	R	-
PmVP161_0258	*gapA*	1005	5	9	0.005525	Glyceraldehyde-3-phosphate dehydrogenase A	G	+
PmVP161_0260	*gmk*	627	2	2	0.00354	Guanylate kinase	F	+
PmVP161_0262	*spoT*	2124	14	1248	0.007322	Bifunctional (p)ppGpp synthase/hydrolase SpoT	K, T	+
PmVP161_0265	*murI*	819	1	1	0.001355	Glutamate racemase	M	+
PmVP161_0279	PmVP161_0279	85	0	0	0	tRNA-Leu	S	-
PmVP161_0283	*rpsO*	270	1	2	0.004115	30S ribosomal protein S15	J	+
PmVP161_0284	*apbC*	1113	9	30	0.008982	Iron-sulfur cluster carrier protein	D	+
PmVP161_0285	*metG*	2049	4	87	0.002168	Methionine--tRNA ligase	J	+
PmVP161_0298	*iscS*	1215	2	3	0.001828	Cysteine desulfurase IscS	H	+
PmVP161_0299	*iscU*	387	1	1	0.002865	Iron-sulfur cluster assembly scaffold protein IscU	C	+
PmVP161_0300	*iscA*	324	0	0	0	Iron-binding protein IscA	C	+
PmVP161_0301	*hscB*	519	1	1	0.002137	Co-chaperone protein HscB	O	+
PmVP161_0302	*hscA*	1863	4	5	0.002385	Chaperone protein HscA	O	+
PmVP161_0303	*fdx*	336	1	1	0.0033	2Fe-2S ferredoxin	C	+
PmVP161_0310	PmVP161_0310	765	9	12	0.013062	hypothetical protein	S	+
PmVP161_0313	*nadK*	918	1	1	0.001209	NAD kinase	F	+
PmVP161_0314	*grpE*	594	0	0	0	Protein GrpE	O	+
PmVP161_0319	*fabB*	1221	6	7	0.00546	3-oxoacyl-(acyl-carrier-protein) synthase 1	I, Q	+
PmVP161_0326	*ispB*	993	8	10	0.008949	Octaprenyl diphosphate synthase	H	+
PmVP161_0327	*rplU*	312	0	0	0	50S ribosomal protein L21	J	+
PmVP161_0328	*rpmA*	258	0	0	0	50S ribosomal protein L27	J	+
PmVP161_0331	*obgE*	1173	2	3	0.001894	GTPase ObgE/CgtA	R	+

PmVP161_0332	*fur*	441	4	9	0.010076	Ferric uptake regulation protein	K	+
PmVP161_0333	*fldA*	525	3	6	0.006342	Flavodoxin 1	C	+
PmVP161_0334	*ybfE*	285	1	2	0.003891	putative protein YbfE	R	-
PmVP161_0336	*seqA*	663	6	63	0.01005	Negative modulator of initiation of replication	L	+
PmVP161_0339	*aroC*	1074	5	14	0.005171	Chorismate synthase	E	+
PmVP161_0340	*mepA*	876	6	79	0.007605	Penicillin-insensitive murein endopeptidase	M	-
PmVP161_0342	*lpxM*	951	0	0	0	Lipid A biosynthesis myristoyltransferase	M	+
PmVP161_0344	PmVP161_0344	2145	5	7	0.002589	hypothetical protein	L	+
PmVP161_0349	*parC*	2253	8	11	0.003945	DNA topoisomerase 4 subunit A	L	+
PmVP161_0350	*parE*	1899	4	4	0.002339	DNA topoisomerase 4 subunit B	L	+
PmVP161_0353	PmVP161_0353	915	9	76	0.010922	hypothetical protein	O	-
PmVP161_0362	*rsxE*	729	2	3	0.003044	Electron transport complex subunit RsxE	C	+
PmVP161_0363	*rsxG*	609	3	3	0.005464	Electron transport complex subunit RsxG	C	+
PmVP161_0364	*rsxD*	1050	2	2	0.002116	Electron transport complex subunit RsxD	C	+
PmVP161_0365	*rsxC*	2508	10	61	0.004429	Electron transport complex subunit RsxC	C	+
PmVP161_0366	*rsxB*	597	5	139	0.009294	Electron transport complex subunit RsxB	C	+
PmVP161_0367	*rsxA*	579	4	791	0.007663	Electron transport complex subunit RsxA	C	+
PmVP161_0368	PmVP161_0368	1056	1	2	0.001052	hypothetical protein	M	+
PmVP161_0374	*mgsA*	459	4	180	0.009662	Methylglyoxal synthase	G	+
PmVP161_0381	*tusE*	330	1	1	0.003367	Sulfurtransferase TusE	P	+
PmVP161_0383	PmVP161_0383	90	0	0	0	tRNA-Ser	S	-
PmVP161_0393	*dnaB*	1404	0	0	0	Replicative DNA helicase	L	+
PmVP161_0394	*alr*	1083	3	6	0.003077	Alanine racemase, biosynthetic	E	+
PmVP161_0397	*pgi*	1650	1	1	0.000673	Glucose-6-phosphate isomerase	G	+
PmVP161_0410	PmVP161_0410	76	0	0	0	tRNA-Asn	S	-
PmVP161_0419	*rlmE*	630	2	3	0.003527	Ribosomal RNA large subunit methyltransferase E	J	+
PmVP161_0420	*ftsH*	1920	3	4	0.001736	ATP-dependent zinc metalloprotease FtsH	O	+
PmVP161_0421	*folP*	828	4	5	0.005362	Dihydropteroate synthase	H	+
PmVP161_0422	*glmM*	1335	4	7	0.003328	Phosphoglucosamine mutase	G	+
PmVP161_0424	PmVP161_0424	690	3	6	0.004831	hypothetical protein	M	-
PmVP161_0440	*erpA*	342	0	0	0	Iron-sulfur cluster insertion protein ErpA	C	+
PmVP161_0441	*map*	804	3	3	0.004144	Methionine aminopeptidase	E	+
PmVP161_0444	*hemL*	1284	4	5	0.00346	Glutamate-1-semialdehyde 2,1-aminomutase	H	+
PmVP161_0451	PmVP161_0451	76	0	0	0	tRNA-Glu	S	-
PmVP161_0466	*queF*	927	9	258	0.010778	NADPH-dependent 7-cyano-7-deazaguanine reductase	R	+
PmVP161_0474	*fabA*	531	2	2	0.004184	3-hydroxydecanoyl-(acyl-carrier-protein) dehydratase	I	+
PmVP161_0478	PmVP161_0478	93	0	0	0	hypothetical protein	S	-
PmVP161_0486	PmVP161_0486	507	1	1	0.002188	hypothetical protein	R	-
PmVP161_0487	PmVP161_0487	477	5	133	0.011628	hypothetical protein	S	-
PmVP161_0491	PmVP161_0491	444	4	126	0.01	hypothetical protein	S	-
PmVP161_0499	PmVP161_0499	786	8	111	0.011299	hypothetical protein	M	+
PmVP161_0506	*recB*	3693	41	459	0.012335	RecBCD enzyme subunit RecB	L	+
PmVP161_0510	*rplM*	429	2	2	0.005168	50S ribosomal protein L13	J	+

PmVP161_0511	*rpsI*	393	3	4	0.008475	30S ribosomal protein S9	J	+
PmVP161_0514	PmVP161_0514	546	3	7	0.006098	3-deoxy-D-manno-octulosonate 8-phosphate phosphatase KdsC	R	+
PmVP161_0515	*kpsF*	936	5	9	0.005931	Arabinose 5-phosphate isomerase KpsF	M	+
PmVP161_0517	*tdeA*	1368	7	7	0.005682	Toxin and drug export protein A	M	+
PmVP161_0518	*glnS*	1680	1	1	0.000661	Glutamine--tRNA ligase	J	+
PmVP161_0522	*dxs*	1845	4	5	0.002408	1-deoxy-D-xylulose-5-phosphate synthase	H	+
PmVP161_0523	*ispA*	888	2	2	0.0025	Farnesyl diphosphate synthase	H	+
PmVP161_0544	*argS*	1734	2	3	0.001281	Arginine--tRNA ligase	J	+
PmVP161_0548	*prfA*	1083	7	9	0.007179	Peptide chain release factor RF1	J	+
PmVP161_0551	*kdsA*	855	4	4	0.005195	2-dehydro-3-deoxyphosphooctonate aldolase	M	+
PmVP161_0553	*lolC*	1191	1	1	0.000933	Lipoprotein-releasing system transmembrane protein LolC	M	+
PmVP161_0554	*lolD_2*	684	2	2	0.003247	Lipoprotein-releasing system ATP-binding protein LolD	V	+
PmVP161_0555	*lolE*	1254	3	3	0.002657	Lipoprotein-releasing system transmembrane protein LolE	M	+
PmVP161_0564	*msbA_1*	1731	2	2	0.001284	Lipid A export ATP-binding/permease protein MsbA	V	+
PmVP161_0565	*cydD_1*	1764	6	12	0.003778	ATP-binding/permease protein CydD	V	+
PmVP161_0566	*trxB*	954	7	26	0.008149	Thioredoxin reductase	C	+
PmVP161_0568	*can*	690	4	5	0.006441	Carbonic anhydrase 2	P	+
PmVP161_0585	*thrS*	1932	6	9	0.00345	Threonine--tRNA ligase	J	+
PmVP161_0588	*infC*	465	0	0	0	Translation initiation factor IF-3	J	+
PmVP161_0589	*rpmI*	198	0	0	0	50S ribosomal protein L35	J	+
PmVP161_0590	*rplT*	354	1	2	0.003135	50S ribosomal protein L20	J	+
PmVP161_0594	*mukF*	1326	3	4	0.002513	Chromosome partition protein MukF	D	+
PmVP161_0595	*mukE*	732	3	3	0.004552	Chromosome partition protein MukE	D	+
PmVP161_0596	*mukB*	4482	11	15	0.002727	Chromosome partition protein MukB	D	+
PmVP161_0599	*sbcB*	1428	1	3	0.000778	Exodeoxyribonuclease I	L	+
PmVP161_0602	PmVP161_0602	279	2	21	0.007937	hypothetical protein	S	-
PmVP161_0603	*topA_1*	2607	7	115	0.002983	DNA topoisomerase 1	L	+
PmVP161_0616	*ihfA*	297	1	2	0.003731	Integration host factor subunit alpha	K	+
PmVP161_0617	*pheT*	2388	2	2	0.00093	Phenylalanine--tRNA ligase beta subunit	J	+
PmVP161_0619	*pheS*	984	5	7	0.005643	Phenylalanine--tRNA ligase alpha subunit	J	+
PmVP161_0623	*folC*	1329	5	6	0.004177	Dihydrofolate synthase/folylpolyglutamate synthase	H	+
PmVP161_0624	*accD*	915	1	2	0.001214	Acetyl-coenzyme A carboxylase carboxyl transferase subunit beta	I	+
PmVP161_0627	*rplY*	285	1	2	0.003891	50S ribosomal protein L25	J	+
PmVP161_0628	PmVP161_0628	615	2	3	0.00361	hypothetical protein	R	+
PmVP161_0631	*asnS*	1404	2	3	0.001582	Asparagine--tRNA ligase	J	+
PmVP161_0634	*lpoA*	1713	14	25	0.009079	Penicillin-binding protein activator LpoA	M	+
PmVP161_0635	PmVP161_0635	360	3	85	0.009259	hypothetical protein	L	-
PmVP161_0638	*ribC*	615	1	4	0.001805	Riboflavin synthase	H	+
PmVP161_0641	*pykA*	1440	0	0	0	Pyruvate kinase II	G	+
PmVP161_0642	PmVP161_0642	77	0	0	0	tRNA-Val	S	-

PmVP161_0646	*dapD*	825	6	12	0.008075	2,3,4,5-tetrahydropyridine-2,6-dicarboxylate N-succinyltransferase	E	+
PmVP161_0648	*cysB*	972	5	6	0.005714	HTH-type Kal regulator CysB	K	+
PmVP161_0649	*rluB*	1038	8	58	0.008556	Ribosomal large subunit pseudouridine synthase B	J	+
PmVP161_0660	*ydjA*	555	6	1117	0.012	Putative NAD(P)H nitroreductase YdjA	C	+
PmVP161_0665	*pgpB*	750	0	0	0	Phosphatidylglycerophosphatase B	I	+
PmVP161_0666	*ribA*	660	1	1	0.001684	GTP cyclohydrolase-2	F	+
PmVP161_0672	PmVP161_0672	75	0	0	0	tRNA-Gln	S	-
PmVP161_0673	PmVP161_0673	85	0	0	0	tRNA-Leu	S	-
PmVP161_0674	PmVP161_0674	77	0	0	0	tRNA-Met	S	-
PmVP161_0676	*hemA*	1305	6	8	0.005106	Glutamyl-tRNA reductase	H	+
PmVP161_0685	*folE*	657	1	1	0.001689	GTP cyclohydrolase 1	F	+
PmVP161_0690	PmVP161_0690	198	1	2	0.005587	hypothetical protein	L	-
PmVP161_0696	PmVP161_0696	444	5	26	0.0125	hypothetical protein	R	+
PmVP161_0697	*ackA*	1206	11	41	0.010129	Acetate kinase	F	+
PmVP161_0698	*pta*	2139	19	37	0.009865	Phosphate acetyltransferase	C	+
PmVP161_0702	*tsaB*	720	1	1	0.001543	tRNA threonylcarbamoyladenosine biosynthesis protein TsaB	O	+
PmVP161_0711	*nrdA*	2271	2	2	0.000978	Ribonucleoside-diphosphate reductase 1 subunit alpha	F	+
PmVP161_0714	*nrdB*	1131	1	1	0.000982	Ribonucleoside-diphosphate reductase 1 subunit beta	F	+
PmVP161_0721	*dapB*	813	1	1	0.001366	4-hydroxy-tetrahydrodipicolinate reductase	E	+
PmVP161_0723	*pgpA*	486	5	23	0.011416	Phosphatidylglycerophosphatase A	I	+
PmVP161_0724	*thiL*	993	4	4	0.004474	Thiamine-monophosphate kinase	H	+
PmVP161_0725	*nusB*	435	2	6	0.005102	K antitermination protein NusB	K	+
PmVP161_0726	*ribE*	474	0	0	0	6,7-dimethyl-8-ribityllumazine synthase	H	+
PmVP161_0731	*dnaK*	1905	8	11	0.004665	Chaperone protein DnaK	O	+
PmVP161_0732	PmVP161_0732	399	4	32	0.011111	hypothetical protein	K	-
PmVP161_0735	*dnaJ*	1119	6	15	0.005952	Chaperone protein DnaJ	O	+
PmVP161_0743	*degS*	1029	3	3	0.003236	Serine endoprotease DegS	O	+
PmVP161_0744	*ribD*	1131	7	10	0.006876	Riboflavin biosynthesis protein RibD	H	+
PmVP161_0746	PmVP161_0746	594	7	300	0.013084	hypothetical protein	L	-
PmVP161_0751	*tyrS*	1188	3	5	0.002804	Tyrosine--tRNA ligase	J	+
PmVP161_0753	*rbfA*	399	1	1	0.002778	Ribosome-binding factor A	J	+
PmVP161_0755	*infB*	2502	5	8	0.00222	Translation initiation factor IF-2	J	+
PmVP161_0756	*nusA*	1485	3	5	0.002244	K termination/antitermination protein NusA	K	+
PmVP161_0758	PmVP161_0758	77	0	0	0	tRNA-Met	S	-
PmVP161_0790	*hemH*	981	1	2	0.001133	Ferrochelatase	H	+
PmVP161_0803	*lapB*	1188	5	7	0.004673	Lipopolysaccharide assembly protein B	G	+
PmVP161_0804	*lapA*	297	1	1	0.003731	Lipopolysaccharide assembly protein A	R	+
PmVP161_0805	*ihfB*	285	2	2	0.007782	Integration host factor subunit beta	K	+
PmVP161_0806	*rpsA*	1650	3	4	0.00202	30S ribosomal protein S1	J	+
PmVP161_0807	*cmk*	684	1	1	0.001623	Cytidylate kinase	F	+
PmVP161_0824	*valS*	2865	7	12	0.002714	Valine--tRNA ligase	J	+

PmVP161_0827	PmVP161_0827	453	3	30	0.007353	hypothetical protein	S	+
PmVP161_0833	*grxA*	264	2	17	0.008403	Glutaredoxin 1	O	+
PmVP161_0843	*serC*	1083	8	10	0.008205	Phosphoserine aminotransferase	E	+
PmVP161_0847	*gyrA*	2682	10	15	0.004143	DNA gyrase subunit A	L	+
PmVP161_0861	PmVP161_0861	87	0	0	0	tRNA-Leu	S	-
PmVP161_0863	*pgsA*	558	3	3	0.005964	CDP-diacylglycerol--glycerol-3-phosphate 3-phosphatidyltransferase	I	+
PmVP161_0865	*kdsB*	777	1	1	0.001429	3-deoxy-manno-octulosonate cytidylyltransferase	M	+
PmVP161_0866	PmVP161_0866	183	0	0	0	hypothetical protein	E, G	+
PmVP161_0867	*lpxK*	978	3	3	0.003405	Tetraacyldisaccharide 4'-kinase	F	+
PmVP161_0868	*msbA_2*	1749	5	12	0.003175	Lipid A export ATP-binding/permease protein MsbA	V	+
PmVP161_0870	*dksA*	438	4	30	0.010127	RNA polymerase-binding K factor DksA	T	+
PmVP161_0872	*folK*	498	1	2	0.002227	2-amino-4-hydroxy-6- hydroxymethyldihydropteridine pyrophosphokinase	H	+
PmVP161_0879	*hns*	405	1	1	0.00274	DNA-binding protein H-NS	R	+
PmVP161_0881	*nagA*	1137	6	12	0.005859	N-acetylglucosamine-6-phosphate deacetylase	G	+
PmVP161_0882	*nagB*	804	9	65	0.012431	Glucosamine-6-phosphate deaminase	G	+
PmVP161_0892	*lpxL*	942	0	0	0	Lipid A biosynthesis lauroyltransferase	M	+
PmVP161_0900	*lpdA*	1419	5	8	0.003912	Dihydrolipoyl dehydrogenase	C	+
PmVP161_0901	*aceF*	1899	3	4	0.001754	Dihydrolipoyllysine-residue acetyltransferase component of pyruvate dehydrogenase complex	C	+
PmVP161_0902	*aceE*	2664	12	13	0.005004	Pyruvate dehydrogenase E1 component	C	+
PmVP161_0903	*crr*	501	5	151	0.011086	PTS system glucose-specific EIIA component	G	+
PmVP161_0906	*rsgA*	1053	3	4	0.003165	Small ribosomal subunit biogenesis GTPase RsgA	R	+
PmVP161_0907	*orn*	555	0	0	0	Oligoribonuclease	A	+
PmVP161_0910	*tsaE*	498	3	4	0.006682	tRNA threonylcarbamoyladenosine biosynthesis protein TsaE	R	+
PmVP161_0911	PmVP161_0911	1230	6	15	0.00542	hypothetical protein	M	+
PmVP161_0913	*miaA*	951	8	89	0.009346	tRNA dimethylallyltransferase	F	+
PmVP161_0922	*sapD*	1050	7	23	0.007407	Peptide transport system ATP-binding protein SapD	P	+
PmVP161_0932	*guaB*	1464	14	46	0.010622	Inosine-5'-monophosphate dehydrogenase	F	+
PmVP161_0934	*guaA*	1572	5	6	0.003534	GMP synthase (glutamine-hydrolyzing)	F	+
PmVP161_0936	*accA*	954	0	0	0	Acetyl-coenzyme A carboxylase carboxyl transferase subunit alpha	I	+
PmVP161_0939	*tilS*	1296	4	6	0.003428	tRNA(Ile)-lysidine synthase	J	+
PmVP161_0944	*adk*	645	2	4	0.003442	Adenylate kinase	F	+
PmVP161_0959	*proQ*	633	7	20	0.012281	RNA chaperone ProQ	T	-
PmVP161_0969	*serS*	1287	3	5	0.002588	Serine--tRNA ligase	J	+
PmVP161_0971	*lolA*	618	2	2	0.003591	Outer-membrane lipoprotein carrier protein	M	+
PmVP161_0972	PmVP161_0972	2793	31	185	0.012331	hypothetical protein	D	+
PmVP161_0977	PmVP161_0977	681	1	8	0.001631	hypothetical protein	P	+
PmVP161_0978	PmVP161_0978	1263	3	3	0.002639	hypothetical protein	P	+
PmVP161_0980	*cca*	1236	7	10	0.006289	Multifunctional CCA protein	F	+

PmVP161_0981	*lolB*	618	5	8	0.008977	Outer-membrane lipoprotein LolB	M	+
PmVP161_0982	*ispE*	888	3	3	0.00375	4-diphosphocytidyl-2-C-methyl-D-erythritol kinase	F	+
PmVP161_0983	*prs*	948	1	2	0.001171	Ribose-phosphate pyrophosphokinase	F	+
PmVP161_0984	PmVP161_0984	1596	11	15	0.007655	hypothetical protein	M	+
PmVP161_0986	*znuB*	786	7	15	0.009887	High-affinity zinc uptake system membrane protein ZnuB	P	+
PmVP161_0987	*ddpF*	981	6	32	0.006795	putative D,D-dipeptide transport ATP-binding protein DdpF	P	+
PmVP161_0988	*dppD*	999	9	42	0.01	Dipeptide transport ATP-binding protein DppD	P	+
PmVP161_0990	*dppB*	1005	9	29	0.009945	Dipeptide transport system permease protein DppB	P	+
PmVP161_0999	*yajC*	297	3	6	0.011194	Sec translocon accessory complex subunit YajC	U	+
PmVP161_1000	*secD*	1851	8	10	0.004802	Protein translocase subunit SecD	U	+
PmVP161_1001	*secF*	972	5	10	0.005714	Protein translocase subunit SecF	U	+
PmVP161_1002	*glyA*	1263	3	3	0.002639	Serine hydroxymethyltransferase	E	+
PmVP161_1023	*secG*	348	2	2	0.006369	Protein-export membrane protein SecG	U	+
PmVP161_1026	*ybaB*	330	3	21	0.010101	Nucleoid-associated protein YbaB	R	+
PmVP161_1034	*lptG*	1071	2	3	0.002075	Lipopolysaccharide export system permease protein LptG	R	+
PmVP161_1035	*lptF*	1107	1	2	0.001003	Lipopolysaccharide export system permease protein LptF	R	+
PmVP161_1037	*mtnN*	690	6	12	0.009662	5'-methylthioadenosine/S-adenosylhomocysteine nucleosidase	F	+
PmVP161_1041	*prfB*	963	4	7	0.004614	Peptide chain release factor RF2	J	+
PmVP161_1042	*lysU*	1506	1	2	0.000737	Lysine--tRNA ligase, heat inducible	J	+
PmVP161_1049	*fabI*	783	3	4	0.004255	Enoyl-(acyl-carrier-protein) reductase (NADH) FabI	I	+
PmVP161_1051	*murA*	1278	2	2	0.001738	UDP-N-acetylglucosamine 1-carboxyvinyltransferase	M	+
PmVP161_1053	PmVP161_1053	378	3	58	0.008798	hypothetical protein	R	+
PmVP161_1058	*lptC*	579	2	3	0.003831	Lipopolysaccharide export system protein LptC	R	+
PmVP161_1059	*lptA*	513	2	3	0.004329	Lipopolysaccharide export system protein LptA	R	+
PmVP161_1060	*lptB*	726	1	1	0.001529	Lipopolysaccharide export system ATP-binding protein LptB	R	+
PmVP161_1065	*rnfH*	300	1	2	0.003704	Protein RnfH	R	+
PmVP161_1067	*pth*	585	3	48	0.005693	Peptidyl-tRNA hydrolase	J	+
PmVP161_1083	*lpxC*	918	4	6	0.004837	UDP-3-O-acyl-N-acetylglucosamine deacetylase	M	+
PmVP161_1084	*ftsZ*	1305	4	4	0.003404	Cell division protein FtsZ	D	+
PmVP161_1085	*ftsA*	1281	2	3	0.001735	Cell division protein FtsA	D	+
PmVP161_1086	*ftsQ*	777	3	4	0.004286	Cell division protein FtsQ	D	+
PmVP161_1087	*ddlB*	930	3	4	0.003584	D-alanine--D-alanine ligase B	F	+
PmVP161_1088	*murC*	1449	3	3	0.002299	UDP-N-acetylmuramate--L-alanine ligase	M	+
PmVP161_1089	*murG*	1065	3	3	0.003128	UDP-N-acetylglucosamine--N-acetylmuramyl-(pentapeptide) pyrophosphoryl-undecaprenol N-acetylglucosamine transferase	M	+
PmVP161_1090	*ftsW*	1191	3	4	0.002799	putative peptidoglycan glycosyltransferase FtsW	M	+
PmVP161_1091	*murD*	1305	6	9	0.005106	UDP-N-acetylmuramoylalanine--D-glutamate ligase	M	+
PmVP161_1092	*mraY*	1083	5	7	0.005128	Phospho-N-acetylmuramoyl-pentapeptide- transferase	M	+

PmVP161_1093	*murF*	1383	2	3	0.001606	UDP-N-acetylmuramoyl-tripeptide--D-alanyl-D- alanine ligase	M	+
PmVP161_1094	*murE*	1485	4	5	0.002992	UDP-N-acetylmuramoyl-L-alanyl-D-glutamate--2, 6-diaminopimelate ligase	M	+
PmVP161_1095	*ftsI*	1806	9	15	0.005535	Peptidoglycan D,D-transpeptidase FtsI	M	+
PmVP161_1096	*ftsL*	318	2	3	0.006969	Cell division protein FtsL	D	+
PmVP161_1115	PmVP161_1115	723	1	1	0.001536	hypothetical protein	H	+
PmVP161_1126	*dnaQ*	762	6	7	0.008746	DNA polymerase III subunit epsilon	L	+
PmVP161_1129	*der*	1533	2	3	0.001449	GTPase Der	R	+
PmVP161_1132	*plsC*	726	2	3	0.003058	1-acyl-sn-glycerol-3-phosphate acyltransferase	I	+
PmVP161_1133	*lpxH*	720	4	4	0.006173	UDP-2,3-diacylglucosamine hydrolase	R	+
PmVP161_1146	*yacG*	204	2	2	0.01087	DNA gyrase inhibitor YacG	R	-
PmVP161_1147	*coaE*	621	7	10	0.012522	Dephospho-CoA kinase	F	+
PmVP161_1153	*rppH*	597	3	15	0.005576	RNA pyrophosphohydrolase	L	+
PmVP161_1155	*lgt*	813	2	3	0.002732	Prolipoprotein diacylglyceryl transferase	M	+
PmVP161_1156	*thyA*	852	2	3	0.002608	Thymidylate synthase	F	+
PmVP161_1157	*tadA*	501	2	3	0.004435	tRNA-specific adenosine deaminase	F, J	+
PmVP161_1162	PmVP161_1162	351	1	1	0.003165	Purine nucleoside phosphoramidase	F, G	+
PmVP161_1166	*pfkA*	966	3	4	0.003448	ATP-dependent 6-phosphofructokinase isozyme 1	F	+
PmVP161_1172	*lepA*	1797	17	113	0.010507	Elongation factor 4	M	+
PmVP161_1173	*lepB*	1023	2	2	0.002172	Signal peptidase I	U	+
PmVP161_1174	*rnc*	678	4	6	0.006547	Ribonuclease 3	J	+
PmVP161_1175	*era_1*	921	5	5	0.006031	GTPase Era	R	+
PmVP161_1200	*yafJ*	828	5	113	0.006702	Putative glutamine amidotransferase YafJ	R	+
PmVP161_1204	*dnaE*	3480	10	10	0.003193	DNA polymerase III subunit alpha	L	+
PmVP161_1221	*hda*	702	2	2	0.003165	DnaA regulatory inactivator Hda	L	+
PmVP161_1227	*dsbE_2*	546	6	256	0.012195	Thiol:disulfide interchange protein DsbE	C, O	+
PmVP161_1241	*hisS*	1272	5	6	0.004367	Histidine--tRNA ligase	J	+
PmVP161_1242	*ispG*	1104	3	5	0.003018	4-hydroxy-3-methylbut-2-en-1-yl diphosphate synthase (flavodoxin)	I	+
PmVP161_1243	*rodZ*	957	11	153	0.012761	Cytoskeleton protein RodZ	R	+
PmVP161_1247	*glnB*	339	4	20	0.013072	Nitrogen regulatory protein P-II	K	+
PmVP161_1254	*lpxB*	1179	6	9	0.00565	Lipid-A-disaccharide synthase	M	+
PmVP161_1255	*lpxA*	789	1	3	0.001406	Acyl-(acyl-carrier-protein)--UDP-N- acetylglucosamine O-acyltransferase	M	+
PmVP161_1256	*fabZ*	459	0	0	0	3-hydroxyacyl-(acyl-carrier-protein) dehydratase FabZ	I	+
PmVP161_1257	*lpxD*	1029	1	1	0.001079	UDP-3-O-(3-hydroxymyristoyl)glucosamine N-acyltransferase	M	+
PmVP161_1258	*skp*	582	3	3	0.005725	Outer membrane p25	M	+
PmVP161_1259	*bamA*	2376	15	19	0.007013	Outer membrane protein assembly factor BamA	M	+
PmVP161_1260	*rseP*	1329	9	12	0.007519	Regulator of sigma-E protease RseP	M	+
PmVP161_1261	*cdsA*	870	2	3	0.002554	Phosphatidate cytidylyltransferase	I	+

PmVP161_1262	*uppS*	723	5	6	0.00768	Ditrans,polycis-undecaprenyl-diphosphate synthase ((2E,6E)-farnesyl-diphosphate specific)	H	+
PmVP161_1263	*dxr*	1218	8	18	0.007293	1-deoxy-D-xylulose 5-phosphate reductoisomerase	I	+
PmVP161_1264	*frr*	558	2	3	0.003976	Ribosome-recycling factor	J	+
PmVP161_1265	*pyrH*	729	5	8	0.00761	Uridylate kinase	F	+
PmVP161_1266	*tsf*	849	2	3	0.002614	Elongation factor Ts	J	+
PmVP161_1267	*rpsB*	723	1	1	0.001536	30S ribosomal protein S2	J	+
PmVP161_1299	*ssb*	501	0	0	0	Single-stranded DNA-binding protein	L	+
PmVP161_1300	*folD*	855	2	2	0.002597	Bifunctional protein FolD protein	F	+
PmVP161_1301	PmVP161_1301	77	0	0	0	tRNA-Pro	S	-
PmVP161_1302	PmVP161_1302	77	0	0	0	tRNA-Arg	S	-
PmVP161_1304	*lipA*	963	4	5	0.004614	Lipoyl synthase	H	+
PmVP161_1305	*lipB*	657	6	9	0.010135	Octanoyltransferase	H	+
PmVP161_1306	PmVP161_1306	297	1	2	0.003731	hypothetical protein	R	+
PmVP161_1314	*rho*	1263	5	6	0.004398	K termination factor Rho	K	+
PmVP161_1317	*acpP*	231	0	0	0	Acyl carrier protein	I, Q	+
PmVP161_1318	*fabG*	729	2	2	0.003044	3-oxoacyl-(acyl-carrier-protein) reductase FabG	I, Q	+
PmVP161_1319	*fabD*	939	4	7	0.004728	Malonyl CoA-acyl carrier protein transacylase	I	+
PmVP161_1320	*fabH*	954	1	1	0.001164	3-oxoacyl-(acyl-carrier-protein) synthase 3	I	+
PmVP161_1322	*rpmF*	171	1	1	0.006494	50S ribosomal protein L32	J	+
PmVP161_1323	*yceD*	525	0	0	0	Large ribosomal RNA subunit accumulation protein YceD	R	+
PmVP161_1335	*psd*	888	2	4	0.0025	Phosphatidylserine decarboxylase proenzyme	I	+
PmVP161_1337	PmVP161_1337	324	3	21	0.010274	hypothetical protein	S	-
PmVP161_1341	*folA*	495	0	0	0	Dihydrofolate reductase	H	+
PmVP161_1343	PmVP161_1343	213	0	0	0	hypothetical protein	P	+
PmVP161_1360	PmVP161_1360	864	9	387	0.011568	hypothetical protein	R	+
PmVP161_1361	*ygfZ*	885	6	8	0.007528	tRNA-modifying protein YgfZ	R	+
PmVP161_1363	*pyrG*	1629	15	21	0.010225	CTP synthase	F	+
PmVP161_1364	*eno*	1302	5	11	0.004266	Enolase	G	+
PmVP161_1365	*yqgF*	423	1	1	0.002625	Putative pre-16S rRNA nuclease	J	+
PmVP161_1373	*fbaA_1*	1080	4	6	0.004115	Fructose-bisphosphate aldolase class 2	G	+
PmVP161_1374	*pgk*	1164	3	3	0.002863	Phosphoglycerate kinase	F	+
PmVP161_1376	PmVP161_1376	261	1	1	0.004255	putative ferredoxin-like protein	C	+
PmVP161_1378	*pssA*	1377	5	7	0.004032	CDP-diacylglycerol--serine O-phosphatidyltransferase	I	+
PmVP161_1392	*srmB*	1329	7	10	0.005848	ATP-dependent RNA helicase SrmB	J, K, L	+
PmVP161_1399	*hemN_1*	1368	3	3	0.002435	Oxygen-independent coproporphyrinogen III oxidase	H	+
PmVP161_1420	*hemD*	747	2	2	0.002972	Uroporphyrinogen-III synthase	H	+
PmVP161_1421	*hemC*	939	2	6	0.002364	Porphobilinogen deaminase	H	+
PmVP161_1424	*tama*	1761	7	14	0.004416	Translocation and assembly module subunit TamA	M	+
PmVP161_1425	*tamB*	3903	17	25	0.004839	Translocation and assembly module subunit TamB	R	+
PmVP161_1427	*glmU*	1377	4	4	0.003226	Bifunctional protein GlmU	M	+
PmVP161_1445	*rpoE*	576	2	3	0.003854	ECF RNA polymerase sigma-E factor	K	+
PmVP161_1446	*rseA*	591	1	6	0.00188	Anti-sigma-E factor RseA	T	+

PmVP161_1448	*rseC*	432	3	5	0.007712	Protein RseC	T	-
PmVP161_1453	PmVP161_1453	95	1	1	0.011628	tRNA-Sec	S	-
PmVP161_1475	*coaA*	951	8	12	0.009346	Pantothenate kinase	F	+
PmVP161_1476	PmVP161_1476	76	0	0	0	tRNA-Thr	S	-
PmVP161_1477	PmVP161_1477	85	0	0	0	tRNA-Tyr	S	-
PmVP161_1478	PmVP161_1478	75	0	0	0	tRNA-Gly	S	-
PmVP161_1479	PmVP161_1479	76	0	0	0	tRNA-Thr	S	-
PmVP161_1502	*proS*	1716	10	15	0.006472	Proline--tRNA ligase	J	+
PmVP161_1518	*ribB*	645	1	1	0.001721	3,4-dihydroxy-2-butanone 4-phosphate synthase	H	+
PmVP161_1522	*rplQ*	390	1	1	0.002849	50S ribosomal protein L17	J	+
PmVP161_1523	*rpoA*	990	1	2	0.001122	DNA-directed RNA polymerase subunit alpha	K	+
PmVP161_1524	*rpsD*	621	4	5	0.007156	30S ribosomal protein S4	J	+
PmVP161_1525	*rpsK*	390	1	3	0.002849	30S ribosomal protein S11	J	+
PmVP161_1526	*rpsM*	357	1	1	0.003106	30S ribosomal protein S13	J	+
PmVP161_1527	*rpmJ*	114	0	0	0	50S ribosomal protein L36	J	+
PmVP161_1528	*secY*	1326	6	6	0.005025	Protein translocase subunit SecY	U	+
PmVP161_1529	*rplO*	435	0	0	0	50S ribosomal protein L15	J	+
PmVP161_1530	*rpmD*	180	0	0	0	50S ribosomal protein L30	J	+
PmVP161_1531	*rpsE*	501	0	0	0	30S ribosomal protein S5	J	+
PmVP161_1532	*rplR*	354	1	1	0.003135	50S ribosomal protein L18	J	+
PmVP161_1533	*rplF*	534	2	2	0.004158	50S ribosomal protein L6	J	+
PmVP161_1534	*rpsH*	393	0	0	0	30S ribosomal protein S8	J	+
PmVP161_1535	*rpsN*	306	2	3	0.007246	30S ribosomal protein S14	J	+
PmVP161_1536	*rplE*	540	1	2	0.002058	50S ribosomal protein L5	J	+
PmVP161_1537	*rplX*	312	0	0	0	50S ribosomal protein L24	J	+
PmVP161_1538	*rplN*	372	1	1	0.002985	50S ribosomal protein L14	J	+
PmVP161_1546	*rpsQ*	255	0	0	0	30S ribosomal protein S17	J	+
PmVP161_1547	*rpmC*	192	0	0	0	50S ribosomal protein L29	J	+
PmVP161_1548	*rplP*	411	0	0	0	50S ribosomal protein L16	J	+
PmVP161_1549	*rpsC*	708	6	9	0.009404	30S ribosomal protein S3	J	+
PmVP161_1550	*rplV*	333	2	4	0.006667	50S ribosomal protein L22	J	+
PmVP161_1551	*rpsS*	276	1	1	0.004016	30S ribosomal protein S19	J	+
PmVP161_1552	*rplB*	822	3	3	0.004054	50S ribosomal protein L2	J	+
PmVP161_1553	*rplW*	303	1	1	0.003663	50S ribosomal protein L23	J	+
PmVP161_1554	*rplD*	603	3	6	0.005525	50S ribosomal protein L4	J	+
PmVP161_1555	*rplC*	630	0	0	0	50S ribosomal protein L3	J	+
PmVP161_1556	*rpsJ*	312	0	0	0	30S ribosomal protein S10	J	+
PmVP161_1571	*cysE*	795	4	4	0.005587	Serine acetyltransferase	E	+
PmVP161_1617	*gyrB*	2421	18	23	0.008261	DNA gyrase subunit B	L	+
PmVP161_1626	*mnmG*	1890	15	16	0.008818	tRNA uridine 5-carboxymethylaminomethyl modification enzyme MnmG	D	+
PmVP161_1630	*atpH_1*	255	2	7	0.008696	ATP synthase subunit c	C	+
PmVP161_1631	*atpF*	471	2	4	0.004717	ATP synthase subunit b	C	+

PmVP161_1634	*atpG*	870	5	19	0.006386	ATP synthase gamma chain	C	+
PmVP161_1635	*atpD*	1374	15	43	0.012126	ATP synthase subunit beta	C	+
PmVP161_1636	*atpC*	429	5	114	0.01292	ATP synthase epsilon chain	C	+
PmVP161_1637	*tusA*	240	0	0	0	Sulfur carrier protein TusA	O	+
PmVP161_1639	*trkH*	1464	12	14	0.009105	Trk system potassium uptake protein TrkH	P	+
PmVP161_1640	*hemG*	513	3	4	0.006494	Protoporphyrinogen IX dehydrogenase (menaquinone)	C, H	+
PmVP161_1653	*gpmA*	684	1	1	0.001623	2,3-bisphosphoglycerate-dependent phosphoglycerate mutase	G	+
PmVP161_1658	*engB*	618	2	3	0.003591	putative GTP-binding protein EngB	D	+
PmVP161_1662	*ybaQ*	327	2	2	0.00678	putative HTH-type Kal regulator YbaQ	K	+
PmVP161_1667	*ftsY*	1380	9	17	0.007246	Signal recognition particle receptor FtsY	D	+
PmVP161_1674	PmVP161_1674	528	6	40	0.012605	hypothetical protein	G	-
PmVP161_1697	*gnd*	1455	8	13	0.006107	6-phosphogluconate dehydrogenase, decarboxylating	H	+
PmVP161_1700	*nfuA*	585	3	4	0.005693	Fe/S biogenesis protein NfuA	C	+
PmVP161_1703	PmVP161_1703	76	0	0	0	tRNA-Glu	S	-
PmVP161_1706	*def*	513	2	4	0.004329	Peptide deformylase	J	+
PmVP161_1707	*fmt*	954	7	13	0.008149	Methionyl-tRNA formyltransferase	J	+
PmVP161_1710	*trkA*	1377	6	6	0.004839	Trk system potassium uptake protein TrkA	P	+
PmVP161_1731	*rpoH*	846	3	3	0.003937	RNA polymerase sigma factor RpoH	K	+
PmVP161_1736	*murB*	1026	7	10	0.007576	UDP-N-acetylenolpyruvoylglucosamine reductase	M	+
PmVP161_1747	*lptD*	2349	11	15	0.005201	LPS-assembly protein LptD	M	+
PmVP161_1755	*ispD*	717	2	3	0.003096	2-C-methyl-D-erythritol 4-phosphate cytidylyltransferase	I	+
PmVP161_1756	*ispF*	477	4	6	0.009302	2-C-methyl-D-erythritol 2,4-cyclodiphosphate synthase	I	+
PmVP161_1769	*trpS*	1002	4	5	0.004435	Tryptophan--tRNA ligase	J	+
PmVP161_1779	*asd*	1110	3	5	0.003003	Aspartate-semialdehyde dehydrogenase	E	+
PmVP161_1798	*thiE*	666	2	3	0.003333	Thiamine-phosphate synthase	H	+
PmVP161_1799	*thiD*	822	2	2	0.002703	Hydroxymethylpyrimidine/phosphomethylpyrimidine kinase	H	+
PmVP161_1800	*thiM*	804	9	16	0.012431	Hydroxyethylthiazole kinase	H	-
PmVP161_1808	*tsaC*	552	2	4	0.004024	Threonylcarbamoyl-AMP synthase	J	+
PmVP161_1825	*alaS*	2625	4	10	0.001693	Alanine--tRNA ligase	J	+
PmVP161_1834	*rpsP*	249	0	0	0	30S ribosomal protein S16	J	+
PmVP161_1835	*rimM*	528	6	12	0.012605	Ribosome maturation factor RimM	J	+
PmVP161_1836	*trmD*	738	1	1	0.001504	tRNA (guanine-N(1)-)-methyltransferase	J	+
PmVP161_1837	*rplS*	351	0	0	0	50S ribosomal protein L19	J	+
PmVP161_1846	*kdtB*	477	1	1	0.002326	Homology to CoaD-Phosphopantetheine adenylyltransferases	F	+
PmVP161_1847	*kdtA*	1284	6	12	0.00519	Bi-functional 3-deoxy-D-manno-octulosonic-acid transferase	M	+
PmVP161_1853	*tpiA*	786	9	18	0.012712	Triosephosphate isomerase	G	+
PmVP161_1857	*corA*	951	2	2	0.002336	Magnesium transport protein CorA	P	+
PmVP161_1872	*nqrA*	1341	4	6	0.003314	Na(+)-translocating NADH-quinone reductase subunit A	C	-
PmVP161_1873	*nqrB*	1233	4	6	0.003604	Na(+)-translocating NADH-quinone reductase subunit B	C	+

PmVP161_1874	*nqrC*	783	3	4	0.004255	Na(+)-translocating NADH-quinone reductase subunit C	C	-
PmVP161_1875	*nqrD*	627	6	7	0.010619	Na(+)-translocating NADH-quinone reductase subunit D	C	+
PmVP161_1876	*nqrE*	597	4	7	0.007435	Na(+)-translocating NADH-quinone reductase subunit E	C	+
PmVP161_1877	*nqrF*	1224	6	10	0.005445	Na(+)-translocating NADH-quinone reductase subunit F	C	+
PmVP161_1878	*apbE*	1047	6	8	0.006363	FAD:protein FMN transferase	H	+
PmVP161_1879	PmVP161_1879	255	1	1	0.004348	hypothetical protein	R	+
PmVP161_1880	*mnmA*	1152	2	2	0.001929	tRNA-specific 2-thiouridylase MnmA	J	+
PmVP161_1896	*tusD*	381	0	0	0	Sulfurtransferase TusD	P	+
PmVP161_1897	*tusC*	360	3	4	0.009259	Protein TusC	P	+
PmVP161_1898	*tusB*	288	2	3	0.007692	Protein TusB	P	-
PmVP161_1899	*rpsL*	375	0	0	0	30S ribosomal protein S12	J	+
PmVP161_1900	*rpsG*	471	1	1	0.002358	30S ribosomal protein S7	J	+
PmVP161_1901	*fusA*	2103	3	5	0.001585	Elongation factor G	J	+
PmVP161_1903	*secE*	411	1	1	0.002703	Protein translocase subunit SecE	U	+
PmVP161_1904	*nusG*	552	4	5	0.008048	K termination/antitermination protein NusG	K	+
PmVP161_1905	*rplK*	429	0	0	0	50S ribosomal protein L11	J	+
PmVP161_1906	*rplA*	690	3	3	0.004831	50S ribosomal protein L1	J	+
PmVP161_1909	*rplJ*	492	0	0	0	50S ribosomal protein L10	J	+
PmVP161_1910	*rplL*	369	0	0	0	50S ribosomal protein L7/L12	J	+
PmVP161_1911	*rpoB*	4029	9	10	0.002481	DNA-directed RNA polymerase subunit beta	K	+
PmVP161_1912	*rpoC*	4254	12	15	0.003134	DNA-directed RNA polymerase subunit beta'	K	+
PmVP161_1914	*hemE*	1065	9	13	0.009385	Uroporphyrinogen decarboxylase	H	+
PmVP161_1915	PmVP161_1915	591	4	83	0.007519	hypothetical protein	R	-
PmVP161_1916	*hupA*	273	1	5	0.004065	DNA-binding protein HU-alpha	L	+
PmVP161_1917	*glmS*	1833	12	93	0.007273	Glutamine--fructose-6-phosphate aminotransferase (isomerizing)	M	+
PmVP161_1918	PmVP161_1918	162	0	0	0	hypothetical protein	R	-
PmVP161_1924	PmVP161_1924	76	0	0	0	tRNA-Glu	S	-
PmVP161_1927	PmVP161_1927	107	0	0	0	5S ribosomal RNA	S	-
PmVP161_1928	PmVP161_1928	77	0	0	0	tRNA-Asp	S	-
PmVP161_1929	PmVP161_1929	76	0	0	0	tRNA-Trp	S	-
PmVP161_1936	*bamD*	783	3	5	0.004255	Outer membrane protein assembly factor BamD	M	+
PmVP161_1940	*ligA*	2022	6	6	0.003297	DNA ligase	L	+
PmVP161_1953	*dapF*	825	5	6	0.006729	Diaminopimelate epimerase	E	+
PmVP161_1959	*folB*	357	1	1	0.003106	Dihydroneopterin aldolase	H	+
PmVP161_1961	*zipA*	972	8	10	0.009143	Cell division protein ZipA	D	+
PmVP161_1964	*hemB*	1026	5	7	0.005411	Delta-aminolevulinic acid dehydratase	H	+
PmVP161_1966	*tatB*	576	3	7	0.00578	Sec-independent protein translocase protein TatB	U	+
PmVP161_1983	*holB*	984	3	4	0.003386	DNA polymerase III subunit delta'	L	+
PmVP161_1984	*tmk*	630	0	0	0	Thymidylate kinase	F	+
PmVP161_1993	*ispH*	945	1	1	0.001175	4-hydroxy-3-methylbut-2-enyl diphosphate reductase	I, M	+
PmVP161_1994	*lspA*	498	0	0	0	Lipoprotein signal peptidase	M, U	+
PmVP161_1995	*ileS*	2799	11	14	0.004365	Isoleucine--tRNA ligase	J	+

PmVP161_1996	*ribF*	936	3	5	0.003559	Riboflavin biosynthesis protein RibF	H	+
PmVP161_1997	*murJ*	1575	2	3	0.00141	putative lipid II flippase MurJ	R	+
PmVP161_1998	*rpsT*	264	1	1	0.004202	30S ribosomal protein S20	J	+
PmVP161_2002	PmVP161_2002	264	3	46	0.012605	hypothetical protein	R	-
PmVP161_2003	PmVP161_2003	77	0	0	0	tRNA-Met	S	-
PmVP161_2020	*rpoD*	1869	7	12	0.004159	RNA polymerase sigma factor RpoD	K	+
PmVP161_2021	*dnaG*	1749	4	8	0.00254	DNA primase	L	+
PmVP161_2022	*rpsU*	216	1	1	0.005128	30S ribosomal protein S21	J	+
PmVP161_2023	*tsaD*	1032	4	6	0.004306	tRNA N6-adenosine threonylcarbamoyltransferase	J	+
PmVP161_2033	*mrcA*	2562	11	14	0.00477	Penicillin-binding protein 1A	M	+
PmVP161_2039	*aroK*	528	6	12	0.012605	Shikimate kinase 1	F	+
PmVP161_2043	PmVP161_2043	312	2	156	0.007117	hypothetical protein	R	-
PmVP161_2044	*secA*	2688	10	13	0.004132	Protein translocase subunit SecA	U	+
PmVP161_2046	PmVP161_2046	267	3	10	0.012448	hypothetical protein	M	-
PmVP161_2047	*holA*	1080	3	3	0.003086	DNA polymerase III subunit delta	L	+
PmVP161_2049	*leuS*	2583	6	7	0.002581	Leucine--tRNA ligase	J	+
PmVP161_2054	*rsmA*	867	9	15	0.011524	Ribosomal RNA small subunit methyltransferase A	J	+
PmVP161_2055	*surA*	933	4	7	0.004762	Chaperone SurA	M	+
PmVP161_2076	*ppa*	528	2	3	0.004202	Inorganic pyrophosphatase	C	+
PmVP161_2084	*ffh*	1377	2	2	0.001613	Signal recognition particle protein	U	+
PmVP161_2085	*plsB*	2430	7	9	0.003201	Glycerol-3-phosphate acyltransferase	I	+
PmVP161_2086	*lexA*	636	6	11	0.010471	LexA repressor	K	+
PmVP161_2087	*rpsF*	378	1	2	0.002933	30S ribosomal protein S6	J	+
PmVP161_2088	*priB*	327	1	1	0.00339	Primosomal replication protein N	L	+
PmVP161_2089	*rpsR*	228	0	0	0	30S ribosomal protein S18	J	+
PmVP161_2090	*rplI*	450	5	30	0.012346	50S ribosomal protein L9	J	+
PmVP161_2092	*glnA*	1419	2	3	0.001565	Glutamine synthetase	E	+
PmVP161_2100	*mnmE*	1359	3	5	0.002451	tRNA modification GTPase MnmE	J	+
PmVP161_2101	*yidC*	1626	8	9	0.005464	Membrane protein insertase YidC	U	+
PmVP161_2103	*rnpA*	327	0	0	0	Ribonuclease P protein component	J	+
PmVP161_2104	*rpmH*	135	0	0	0	50S ribosomal protein L34	J	+

[a] COG groups are as follows. A, RNA processing and modification; C, Energy production and conversion; RNA processing and modification; D, Cell cycle control, cell division, chromosome partitioning; E, Amino acid transport and metabolism; F, Nucleotide transport and metabolism; G, Carbohydrate transport and metabolism; H, Coenzyme transport and metabolism; I, Lipid transport and metabolism; J, Translation, ribosomal structure and biogenesis; J, Translation, ribosomal structure and biogenesis; J, Translation, ribosomal structure and biogenesis; K, Transcription; L, Replication, recombination and repair; M, Cell wall/membrane/envelope biogenesis; O, Post-translational modification, protein turnover, and chaperones; P, Inorganic ion transport and metabolism; R, Function Unknown; S, No predicted group; T, Signal transduction mechanisms; U, Intracellular trafficking, secretion, and vesicular transport; V, Defense mechanisms.

Table S4. Oligonucleotides used in this study

Oligonucleotide	Sequence (5'-3')	Description
**TraDIS analysis**		
BAP3453	CGTCATGGTCTTTGTAGTCTATGG	Outward firing primer that anneals upstream of the *Himar1* right inverted repeat, used to identify *Himar1* insertion sites in the VP161 genome
BAP8034	P-G*ATCGGAAGAGCGGTTCAGCAGGTTTTTTTTTT CAAAAAAA*A	Splinkerette adapter top strand; P- represents a 5' phosphate and * represents a phosphorothioate bond (7)
BAP8035	G*AGATCGGTCTCGGCATTCCTGCTGAACCGCTCT TCCGATC*T	Splinkerette adapter bottom strand; P- represents a 5' phosphate and * represents a phosphorothioate bond (7)
BAP8037	CAAGCAGAAGACGGCATACGAGATTAAGGCGAGA GATCGGTCTCGGCATTCC	Splinkerette adapter-specific oligonucleotide for TraDIS library amplification, contains an index sequence to allow multiplexing of libraries and Illumina P7 sequence at the 5' end
BAP8038	CAAGCAGAAGACGGCATACGAGAT**CGTACTAG**GA GATCGGTCTCGGCATTCC	Splinkerette adapter-specific oligonucleotide for TraDIS library amplification, contains an index sequence (bold) to allow multiplexing of libraries and Illumina P7 sequence at the 5' end
BAP8039	CAAGCAGAAGACGGCATACGAGAT**AGGCAGAA**GA GATCGGTCTCGGCATTCC	Splinkerette adapter-specific oligonucleotide for TraDIS library amplification, contains an index sequence (bold) to allow multiplexing of libraries and Illumina P7 sequence at the 5' end
BAP8040	CAAGCAGAAGACGGCATACGAGAT**TCCTGAGC**GA GATCGGTCTCGGCATTCC	Splinkerette adapter-specific oligonucleotide for TraDIS library amplification, contains an index sequence (bold) to allow multiplexing of libraries and Illumina P7 sequence at the 5' end
BAP8042	GGTTCTAGAGACCGGGGACTTATCAGC	Custom *Himar1* transposon-specific Illumina sequencing oligonucleotide
BAP8043	TTCAGCAGGAATGCCGAGACCGATCTC	Custom adapter index-specific Illumina sequencing oligonucleotide
BAP8265	GTAACATCAGAGGGTACCTCG	Outward-firing Tn*7*-specific primer. Anneals to region upstream of right junction. Used to check, by Sanger sequencing, the position of Tn*7* in TraDIS parent strain VP161Tn*7*
BAP8350	AATGATACGGCGACCACCGAGATCTACACCTCTAG AAAGTATAGGAACTTCGAACCG	*Himar1*-specific oligonucleotide for TraDIS library amplification, contains Illumina P5 at the 5' end
BAP8479	AATGATACGGCGACCACCGAGATCTACACCTGGG GTACGCGTCTAGGCGG	*Himar1*-specific oligonucleotide for TraDIS library amplification, contains Illumina P5 at the 5' end
**TargeTron mutagenesis**		
BAP6544	CGAAATTAGAAACTTGCGTTCAGTAAAC	EBS universal primer used for re-targeting TargeTron intron to target gene
BAP8707	AAAAAAGCTTATAATTATCCTTATGATTCCCCTGCG TGCGCCCAGATAGGGTG	IBS TargeTron oligonucleotide for retargeting group II intron in pAL953 to *hexA*
BAP8708	CAGATTGTACAAATGTGGTGATAACAGATAAGTCC CCTGCCCTAACTTACCTTTCTTTGT	EBS1d TargeTron oligonucleotide for retargeting group II intron in pAL953 to *hexA*

Oligonucleotide	Sequence (5’-3’)	Description
BAP8709	TGAACGCAAGTTTCTAATTTCGGTTAATCATCGATA GAGGAAAGTGTCT	EBS2 TargeTron oligonucleotide for retargeting group II intron in pAL953 to *hexA*
BAP8712	AAAAAAGCTTATAATTATCCTTACGTCACGCCGCA GTGCGCCCAGATAGGGTG	IBS TargeTron oligonucleotide for retargeting group II intron in pAL953 to *ppx*
BAP8713	CAGATTGTACAAATGTGGTGATAACAGATAAGTCG CCGCATTTAACTTACCTTTCTTTGT	EBS1d TargeTron oligonucleotide for retargeting group II intron in pAL953 to *ppx*
BAP8714	TGAACGCAAGTTTCTAATTTCGGTTTGACGTCGAT AGAGGAAAGTGTCT	EBS2 TargeTron oligonucleotide for retargeting group II intron in pAL953 to *ppx*
BAP8960	AAAAAAGCTTATAATTATCCTTACTCGTCCTGCGG GTGCGCCCAGATAGGGTG	IBS TargeTron oligonucleotide for retargeting group II intron in pAL953 to *ptsH*
BAP8961	CAGATTGTACAAATGTGGTGATAACAGATAAGTCC TGCGGCATAACTTACCTTTCTTTGT	EBS1d TargeTron oligonucleotide for retargeting group II intron in pAL953 to *ptsH*
BAP8962	TGAACGCAAGTTTCTAATTTCGATTACGGCTCGAT AGAGGAAAGTGTCT	EBS2 TargeTron oligonucleotide for retargeting group II intron in pAL953 to *ptsH*
BAP9151	AAAAAAGCTTATAATTATCCTTAAGCTCCGGCAAG GTGCGCCCAGATAGGGTG	IBS TargeTron oligonucleotide for retargeting group II intron in pAL953 to *spoT*
BAP9152	CAGATTGTACAAATGTGGTGATAACAGATAAGTCG GCAAGGGTAACTTACCTTTCTTTGT	EBS1d TargeTron oligonucleotide for retargeting group II intron in pAL953 to *spoT*
BAP9153	TGAACGCAAGTTTCTAATTTCGATTGAGCTTCGATA GAGGAAAGTGTCT	EBS2 TargeTron oligonucleotide for retargeting group II intron in pAL953 to *spoT*
**Complementation plasmid construction**		
BAP612	GTAAAACGACGGCCAGT	M13 universal primer for sequencing the cloning region in pAL99 and derivatives
BAP2679	TTGTGTGGAATTGTGAGCGGA	pAL99-specific primer for sequencing the cloning region in pAL99 and derivatives
galU		
BAP9157	TGGCTATCTAGAAGAAGGAAAAAAAATGAAAGCAA TT	Forward flanking *galU* oligonucleotide, includes XmaI site
BAP9158	TAAACAGTCGACTTAAAAACTTTTGACGAGCTG	Reverse flanking *galU* oligonucleotide, includes SalI site
hexA		
BAP9187	TGCAGACCCGGGAGGAGGGATAGAGCCTCGATG	Forward flanking *hexA* oligonucleotide, includes XmaI site
BAP8865	AGCGGGGTCGACTTATTTGGTTTGTAATGCATTAT TA	Reverse flanking *hexA* oligonucleotide, includes SalI site
hyaD		
BAP8868	GTTAATGAGCTCAGGAGGGAAAATGAATACATTAT CACAAGCA	Forward flanking *hyaD* oligonucleotide, includes SacI site

Oligonucleotide	Sequence (5’-3’)	Description
BAP8869	AATGCAGTCGACTTATAGAGTTATACTATTAATAATGA	Reverse flanking *hyaD* oligonucleotide, includes SalI site
BAP8986	TGATGGTAGTCAGGAAGATCTA	*hyaD*-specific oligonucleotide used to sequence complementation plasmid
BAP8987	AGGGTACGCATCATGTCTAAA	*hyaD*-specific oligonucleotide used to sequence complementation plasmid
BAP8988	TATTAATAAATTAAGTCAGTTAAATC	*hyaD*-specific oligonucleotide used to sequence complementation plasmid
pgm		
BAP9162	TTAATATCTAGAAGGAGAATTTATGTCAATCTTT	Forward flanking *pgm* oligonucleotide, includes XmaI site
BAP9070	GCTTAAGTCGACTTAGCAATCCTGCTTACCAA	Reverse flanking *pgm* oligonucleotide, includes SalI site
BAP9186	CTTTTTCTTCTGGATTCGGG	*pgm*-specific oligonucleotide used to sequence complementation plasmid
ppx		
BAP9172	ATTTGACCCGGGAGGAAGAATATGAAGAGTGAAA	Forward flanking *ppx* oligonucleotide, includes XmaI site
BAP8945	TGGGCGGGATCCTTAGTTAAACGTGATATCTAATCC	Reverse flanking *ppx* oligonucleotide, includes BamHI site
BAP9185	TGTCAATGTTGTTGGCACC	*ppx*-specific oligonucleotide used to sequence complementation plasmid
ptsH		
BAP9160	TCTTTGTCTAGAAGGAGGAAAAACATGTACTCAAAAGAC	Forward flanking *ptsH* oligonucleotide, includes XmaI site
BAP9072	GCTAACGTCGACTTATTCTAAGGTTGGAATAAGT	Reverse flanking *ptsH* oligonucleotide, includes SalI site
spoT		
BAP9188	ACATTGCCCGGGAGGAGGTGTCCCTTGTATCTTT	Forward flanking *spoT* oligonucleotide, includes XmaI site
BAP9189	GGAATTGTCGACTCATTGATTGATATTACGTTCA	Reverse flanking *spoT* oligonucleotide, includes SalI site
BAP9233	TCACCACCACGTAAATTTTT	*spoT*-specific oligonucleotide used to sequence complementation plasmid
**qRT-PCR**		
BAP2106	GCCCTTTCCGATAAATTGCAA	Forward *gyrB* oligonucleotide used for qRT-PCR
BAP2107	ATCGCGGCTAATGGTGCTT	Reverse *gyrB* oligonucleotide used for qRT-PCR
BAP9299	GGGGTGGAGAAGATGTGGAA	Forward *hyaD* oligonucleotide used for qRT-PCR
BAP9300	CCTGGTGGCTCTTGATGGTA	Reverse *hyaD* oligonucleotide used for qRT-PCR
BAP9301	TCGCAGTTAGATGGCCAAGA	Forward *fis* oligonucleotide used for qRT-PCR
BAP9302	GCTGCACGAGTTTGATTTCC	Reverse *fis* oligonucleotide used for qRT-PCR

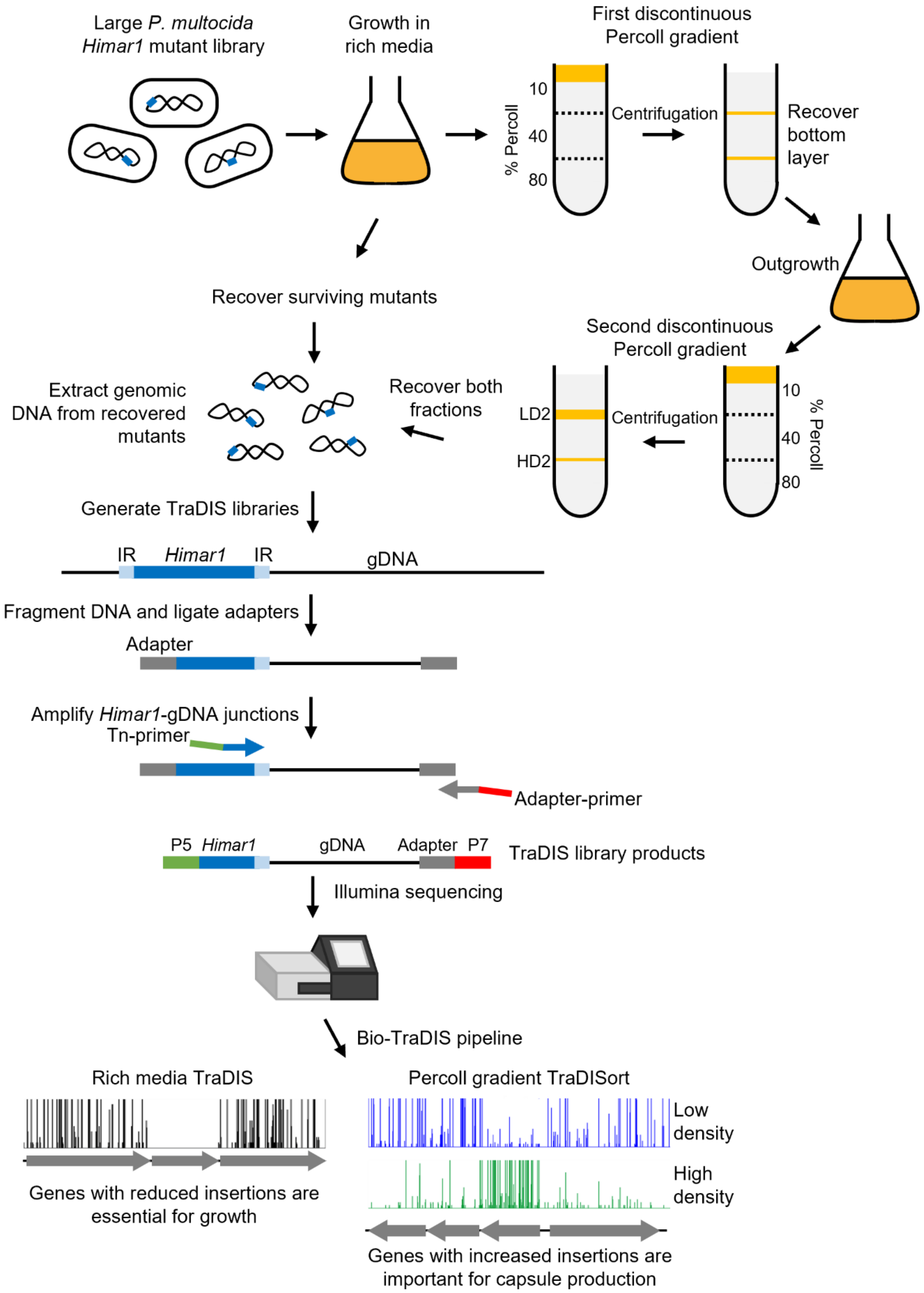

Fig S1. Overview of TraDIS experiments performed in this study. A large, random *P. multocida* mutant library was generated using the *Himar1* transposon. The VP161

Himar1 mutant library was grown in heart infusion broth to mid-exponential growth stage with TraDIS analysis conducted to identify all genes essential for growth in rich media. For TraDISort experiments, the VP161 *Himar1* mutant library was subjected to two consecutive discontinuous 10%, 40%, 80% Percoll discontinuous gradient centrifugation to separate low density cells (capsulated) from high density cells (acapsular). TraDIS libraries representing the LD2 (capsulated) and HD2 (acapsular) cell layer were sequenced via Illumina sequencing, with reads aligned to the VP161 genome to identify *Himar1* insertion sites. Genes from cells derived from the HD2 cell layer with a significantly increased number of Himar1-specific sequence reads, compared to reads generated from cells derived from the LD2 cell layer, were designated important for capsule production. gDNA: genomic DNA. IR: Inverted repeat. Tn: transposon. LD2: Low-density cell layer 2. HD2: High-density cell layer 2.

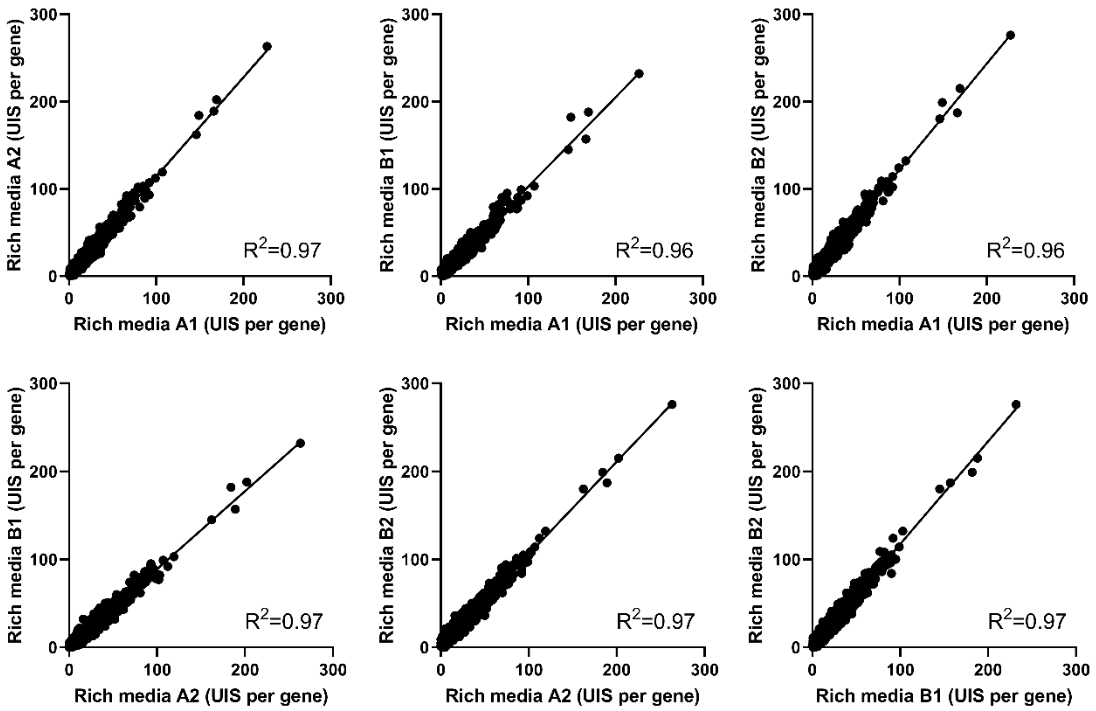

Fig S2. Unique *Himar1* insertion site (UIS) correlation plots. The number of UIS identified in each gene was plotted comparing all four rich media TraDIS libraries A1, A2, B1, B2. The R^2 values were determined by simple linear regression analysis for each of the datasets.

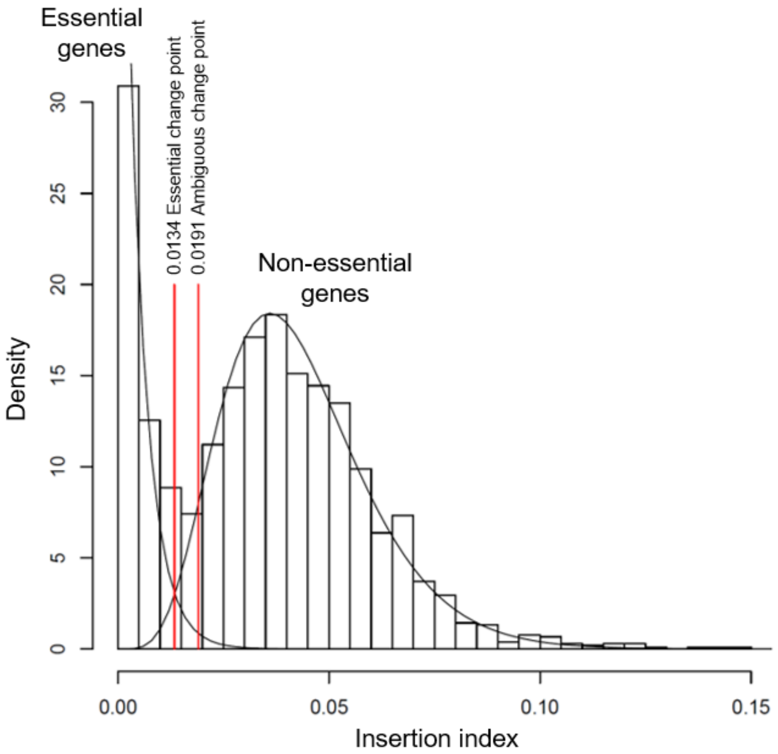

Fig S3. Histogram of insertion indexes for all genes from the four rich media TraDIS libraries (A1, A2, B1, B2) combined. Insertion indexes were calculated for all genes by dividing the number of unique *Himar1* insertion sites by gene length. Normal curves were drawn for the bimodal dataset, with the intersection between the two curves (0.0134) used as the cut-off to call a gene essential for *P. multocida* growth in rich media.

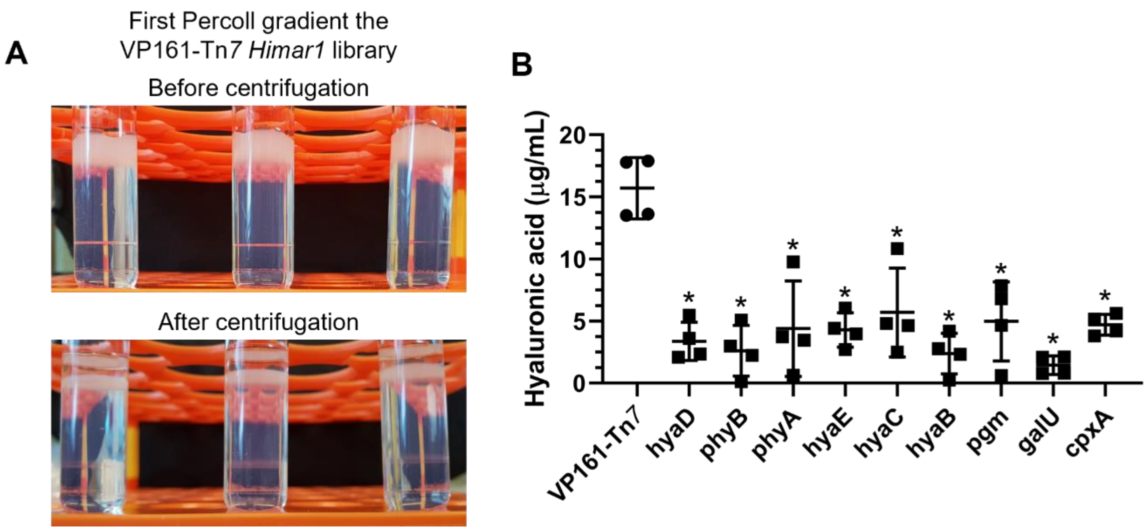

Fig S4. Isolation of acapsular mutants from the large *P. multocida* strain VP161-Tn*7 Himar1* mutant library. **(A)** Separation of the *P. multocida* VP161-Tn*7 Himar1* mutant library using a discontinuous 10%, 40%, 80% Percoll gradient. Following the first gradient centrifugation, cells separated into a top, low density cell layer and a bottom, high density cell layer. Cells were collected from the lower cell layer only and grown to mid-exponential growth phase in HI, before being subjected to a second gradient centrifugation. The top, low density (LD2) layer of cells, containing predominantly capsular mutants, and the bottom, high density (HD2) layer of cells, containing predominantly acapsular mutants, were then used for further study. **(B)** Amount of hyaluronic acid (HA) capsule produced by individual VP161-Tn*7 Himar1* mutants recovered from the HD2 cell layer. HA production was measured in biological quadruplicate at mid-exponential growth phase, and compared to the VP161-Tn*7* parent strain, with the VP161 *hyaD* TargeTron mutant (AL3574) included as a known negative control. Significant difference in capsule production was determined by comparing each mutant to VP161-Tn*7* using a Mann-Whitney U-test; * represents $p < 0.05$ and ** represents $p < 0.01$. Error bars represent mean ± standard deviation.

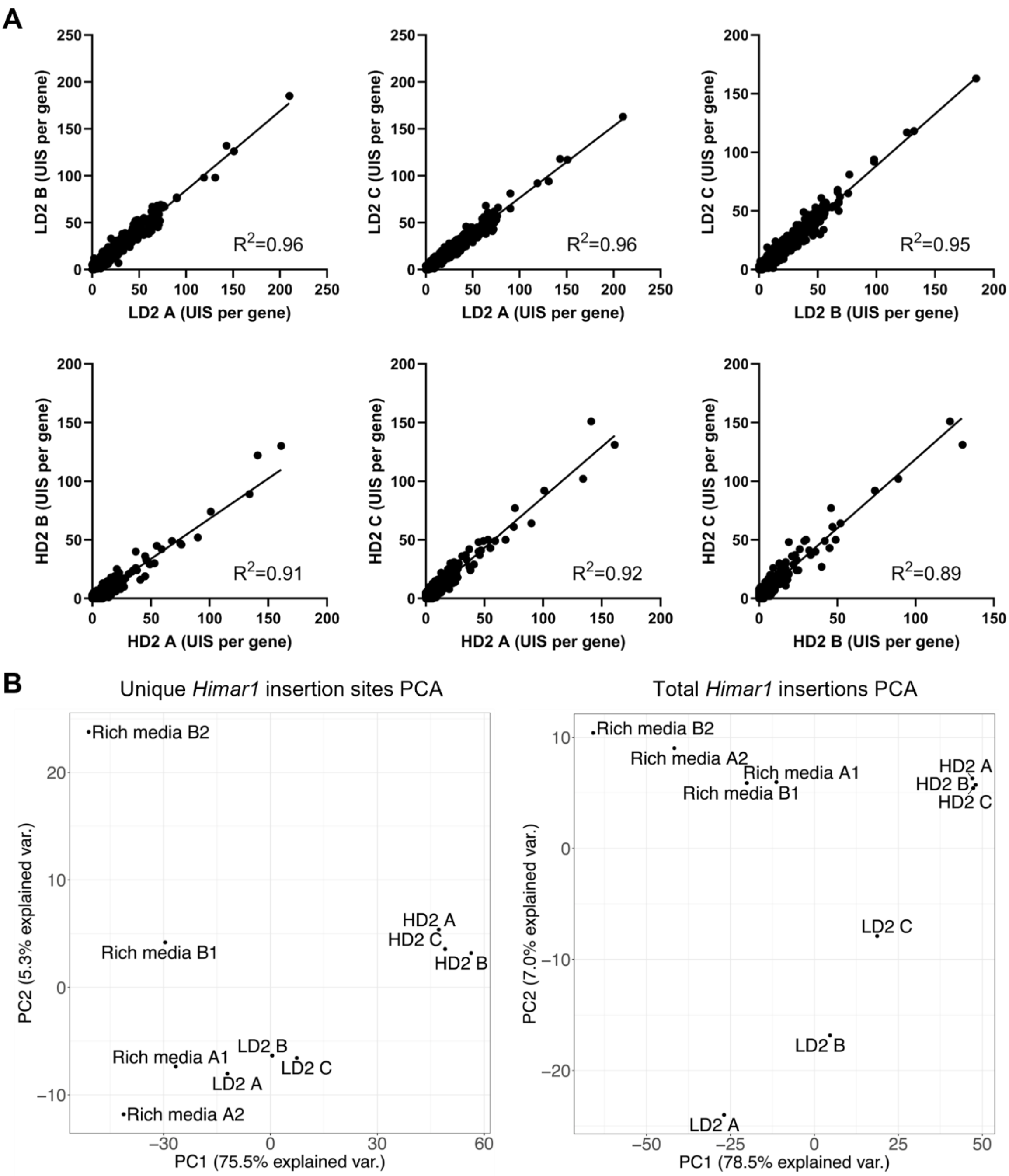

Fig S5. Reproducibility of the TraDISort library replicates. **(A)** Correlation plots showing the number of unique *Himar1* insertion sites (UIS) identified in each gene compared between the LD2 and HD2 library replicates. The R^2 values were determined by simple linear regression analysis for each of the datasets. **(B)** Principal component analysis (PCA) plots for the rich media (A1, A2, B1, B2), LD2 (A, B, C) and HD2 (A, B, C) TraDIS library replicates generated by comparing UIS or total *Himar1* insertions per gene. PCA was performed using the prcomp function in R.
